# New insights on the species-specific allelopathic interactions between macrophytes and marine HAB dinoflagellates

**DOI:** 10.1371/journal.pone.0187963

**Published:** 2017-11-17

**Authors:** Hela Ben Gharbia, Ons Kéfi-Daly Yahia, Philippe Cecchi, Estelle Masseret, Zouher Amzil, Fabienne Herve, Georges Rovillon, Habiba Nouri, Charaf M’Rabet, Douglas Couet, Habiba Zmerli Triki, Mohamed Laabir

**Affiliations:** 1 Research Group on Oceanography and Plankton Ecology, Tunisian National Institute of Agronomy (INAT), IRESA-Carthage University. U.R.13ES36 Marine Biology (University of Tunis El Manar), Tunis, Tunisia; 2 Center for Marine Biodiversity, Exploitation and Conservation (MARBEC): IRD, IFREMER, CNRS, Montpellier University, Montpellier, France; 3 IFREMER-Phycotoxins Laboratory, Nantes, France; 4 Institut de Recherche pour le Développement (IRD), Tunis, Tunisia; University of Connecticut, UNITED STATES

## Abstract

Macrophytes are known to release allelochemicals that have the ability to inhibit the proliferation of their competitors. Here, we investigated the effects of the fresh leaves of two magnoliophytes (*Zostera noltei* and *Cymodocea nodosa*) and thalli of the macroalgae *Ulva rigida* on three HAB-forming benthic dinoflagellates (*Ostreopsis* cf. *ovata*, *Prorocentrum lima*, and *Coolia monotis*). The effects of *C*. *nodosa* and *U*. *rigida* were also tested against the neurotoxic planktonic dinoflagellate *Alexandrium pacificum* Litaker sp. nov (former *Alexandrium catenella*). Co-culture experiments were conducted under controlled laboratory conditions and potential allelopathic effects of the macrophytes on the growth, photosynthesis and toxin production of the targeted dinoflagellates were evaluated. Results showed that *U*. *rigida* had the strongest algicidal effect and that the planktonic *A*. *pacificum* was the most vulnerable species. Benthic dinoflagellates seemed more tolerant to potential allelochemicals produced by macrophytes. Depending on the dinoflagellate/macrophyte pairs and the weight of leaves/thalli tested, the studied physiological processes were moderately to heavily altered. Our results suggest that the allelopathic activity of the macrophytes could influence the development of HAB species.

## Introduction

Allelopathy is a prevalent natural phenomenon in terrestrial and aquatic ecosystems. It is now widely accepted that plants, macrophytes and various microorganisms can produce and release chemicals into the surrounding environment [1–3]. Allelopathy has been extensively studied in terrestrial habitats and harmful effects of plants on other plants or crops are quite well known [4]. The involved allelopathic compounds (allelochemicals) have been explored as natural substitutes of pesticides for pest control [5,6]. In aquatic ecosystems, allelopathy has been more investigated in freshwater environments than in marine habitats [7,8].

Harmful algal blooms (HAB) occur frequently in both freshwater and marine areas, and represent a significant threat to water-supply reservoirs, fisheries, aquaculture, public health, and tourism [9–11]. In order to control and mitigate the proliferation and dispersion of HAB species, different strategies have been adopted [12]. However, the most well known methods (such as the dispersion of flocculant clays [13] or the use of copper sulfate [14]) are still expensive, time-consuming, and may have dangerous environmental consequences.

It has been demonstrated, *in situ*, that microalgae are less abundant in the presence of macrophytes [15,16], which suggests that seaweeds and seagrasses might produce and release allelochemicals acting as natural biological mitigation agents. It has been reported that different macrophytes were able to reduce the proliferation of red tide species with relatively low detrimental effects on the surrounding environment [17–19]. The inhibitory properties of macrophytes are mainly attributed to the action of the released bioactive molecules [2,7,20]. These allelochemicals are mainly secondary metabolites [21] that might be released either actively by exudation from intact living tissue or passively by leaching, leaf wounds or decaying shoots [8,21]. The most well known molecules are phenolic acids, flavonoids, tannins, terpenoids, alkaloids, and various polyunsaturated fatty acids (PUFAs) [7,17].

Knowing the structural diversity of allelochemicals, it is evident that allelopathy must involve more than one mechanism of action. It has been shown that allelochemicals are likely to disturb a variety of physiological processes of the target organisms, such as mitosis, cell division, membrane permeability, ion and water uptake, cell structure and morphology, respiration, photosynthesis, enzyme activity, signal transduction, and protein and nucleic acid synthesis [20,22–25].

Studies on the allelopathic interactions between macrophytes and HAB-forming benthic dinoflagellates are rare [26], since most of the research focuses on the potential effects of allelochemicals on planktonic species. Investigations on the allelopathy exerted by macrophytes on marine benthic dinoflagellates will be of great interest for mitigation purposes. In fact, many of these organisms are emergent HAB species involved in the production of potent toxins, that may threaten both ecosystem functioning and human health [27,28].

Here, we investigated the nature of the allelopathic interactions between three widely distributed macrophytes (*Zostera noltei*, *Cymodocea nodosa*, and *Ulva rigida*) and three HAB-forming marine benthic dinoflagellates (*Ostreopsis* cf. *ovata*, *Prorocentrum lima*, and *Coolia monotis*). The potential allelopathic effects of the magnoliophyte *C*. *nodosa* and the green macroalgae *U*. *rigida* were also tested on the neurotoxic planktonic dinoflagellate *Alexandrium pacificum*, whose sensitivity to *Zostera* spp. allelochemicals has already been demonstrated [29].

The studied benthic dinoflagellates (*O*. cf. *ovata*, *P*. *lima*, and *C*. *monotis*) constitute a significant part of epibenthic assemblages in marine ecosystems worldwide. *O*. cf. *ovata* can produce palytoxin, ovatoxins, and mascarenotoxins [30–34] and is responsible for recurrent toxic blooms in the Mediterranean Sea (up to 1.8 x 10^6^ cells.L^-1^) with notable effects on socio-economic activities and public health [35,36]. *P*. *lima*, a cosmopolitan toxic dinoflagellate, is also known to produce several toxins, such as okadaic acid, dinophysistoxins, prorocentrolide, and prorocentin [37–39], that can cause diarrhetic shellfish poisoning episodes. Recently, a bloom of *P*. *lima* was recorded in Cartagena Bay (Cartagena de Indias, Colombian Caribbean) with cell abundances reaching 2.1 to 4.5 x 10^6^ cells.L^-1^[40]. *C*. *monotis* is a bloom forming species able to reach high cell densities (5 x 10^5^ cells.L^-1^ reported in the North Lake of Tunis-Tunisia [41]), but its toxic properties are not confirmed [34]. The planktonic dinoflagellate *A*. *pacificum* produces potent neurotoxins responsible for paralytic shellfish poisoning syndrome, and is known to induce extensive blooms in marine waters worldwide (up to 1.4 x 10^7^ cells.L^−1^ reported in Thau lagoon, French Mediterranean coast [42]) with disastrous effects on fisheries and aquaculture [43].

The three tested macrophytes are cosmopolitan species. *Z*. *noltei* occurs in European, African, and Atlantic coasts [44]. It has been suggested that the chemical content of *Zostera* species might negatively affect growth and/or photosynthesis of microalgae [29,45–47]. *Cymodocea nodosa* is one of the most important magnoliophytes in the Mediterranean Sea, although data on allelochemicals released by this macrophyte and information about their potential effects on surrounding organisms are very limited [48–50]. *Ulva rigida* is a common green subtidal marine seaweed distributed worldwide. *Ulva* species are ‘‘green tide” forming macroalgae, which are known to efficiently weaken HABs due to their negative allelopathic properties [51–54].

The aim of our study was to examine the potential allelopathic interactions induced by macrophytes (*Z*. *noltei*, *C*. *nodosa*, and *U*. *rigida*) on HAB-forming dinoflagellate species, including benthic (*O*. cf. *ovata*, *P*. *lima*, and *C*. *monotis*) and planktonic (*A*. *pacificum*) microorganisms. Co-culture experiments of each microalgae with fresh macrophyte leaves/thalli were performed through controlled laboratory experiments in microcosms. The allelopathic effects of the tested macrophytes on various physiological processes of the dinoflagellate species including growth, photosynthesis, and toxin production were investigated.

## Material and methods

### Dinoflagellate cultures

Non-axenic monoclonal cultures of the three thermophilic benthic dinoflagellates *Ostreopsis* cf. *ovata* (OOBZT14), *Prorocentrum lima*, (PLBZT14) and *Coolia monotis* (CMBZT14) were grown in enriched natural seawater culture medium (ESNW medium; NO_3_^-^ and PO_4_^3-^ concentrations: 549 μmol.L^-1^ and 22.4 μmol.L^-1^, respectively [55]) at stable conditions (salinity: 36; temperature: 25°C; irradiance: 100 μmol photons.m^-2^.s^-1^ in a 12:12 light:dark cycle). The planktonic dinoflagellate *Alexandrium pacificum* (ABZ1) (former *A*. *catenella*, [56]) was cultured under the same conditions but at a temperature of 20°C, which corresponds to its optimal growth [57]. OOBZT14, PLBZT14, and CMBZT14 strains were isolated from the Bizerte Bay [34] while the ABZ1 strain was obtained from the culture collection of the Center for Marine Biodiversity, Exploitation and Conservation (Montpellier University, France) and was originally isolated from the Bizerte lagoon [58]. Both sites are located in Northern Tunisia, Southern Mediterranean Sea.

### Macrophyte collection site

**Fig 1:**
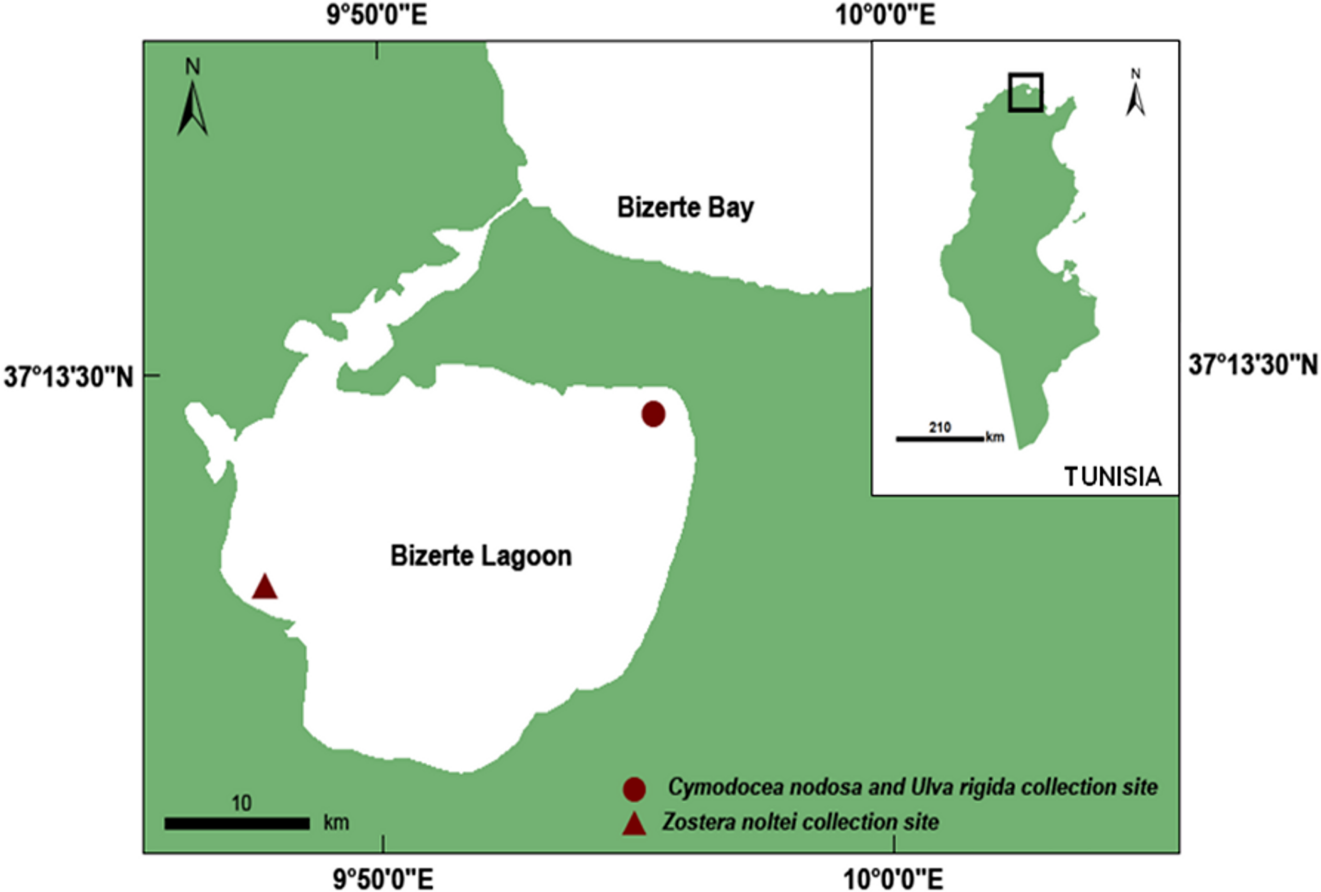
Macrophyte collection sites (North of Tunisia, Southern Mediterranean Sea). Circle: Menzel Jemil station; Triangle: Menzel Bourguiba station.

Fresh leaves/thalli of the three macrophytes *Zostera noltei*, *Cymodocea nodosa* and *Ulva rigida* were collected between April and October 2015 in the Bizerte lagoon (Fig 1). This lagoon is dominated by dense *C*. *nodosa* beds. It is also characterized by *U*. *rigida* mats in its eastern part and by the presence of patchy *Z*. *noltei* meadows in its western part. The collection of macrophyte samples for scientific purposes didn’t require a specific authorization. Macrophytes were carefully gathered to keep belowground parts intact. All samples were placed in plastic boxes containing *in situ* seawater to prevent evaporation, and then immediately transported to the laboratory. Plant material was initially washed with freshwater to remove sand and salt, then carefully cleaned with filtered seawater before being briefly rinsed with distilled water to remove potential attached organisms.

### Dinoflagellate-macrophyte co-incubations

Four different weights of each macrophyte were tested on each dinoflagellate species. For experiments with the two magnoliophytes (*Z*. *noltei* and *C*. *nodosa*), the dinoflagellates were cultured with 0.1 g, 0.3 g, 0.75 g, and 1.5 g fresh weight (FW) of leaves. For experiments with the macroalgae (*U*. *rigida*), 0.08 g, 0.16 g, 0.5 g and 1.0 g FW of thalli were tested. Cleaned fresh leaves/thalli were blotted dry, weighed, and placed in 250 mL culture flasks filled with culture medium. Each flask was inoculated with dinoflagellates in order to obtain an initial concentration of ca. 800–1000 cell.mL^-1^ in a final volume of 200 mL. The obtained concentrations of leaves/thalli were comparable to those observed *in situ* [15,59–61]. All strains were cultured to the exponential phase before inoculation, and all experiments were conducted in triplicate over a time course of 10 days. For each experiment, controls (10-day incubations of the four dinoflagellates in ESNW without macrophytes) were also performed in triplicate. One gram FW of each macrophyte was dried in a drying oven in order to determine the equivalent dry weights (DW) of leaves/thalli. Allelopathic effects of *Z*. *noltei*, *C*. *nodosa*, and *U*. *rigida* fresh leaves/thalli were tested on the three benthic strains, while only *C*. *nodosa* and *U*. *rigida* were tested on the planktonic *A*. *pacificum*.

At the beginning (Day 0) and the end (Day 10) of each experiment, aliquots (15 mL) were taken from each flask. pH and dissolved oxygen were measured with a multiparameter HACH (HQ40d multi) sensor. Samples were then filtered (Whatman GF/F, diameter 47 mm, porosity 0.7 μm) and stored at -20°C for nutrient analysis. Concentrations of the main nutrients (NO_3_^-^ and PO_4_^3-^) were analyzed with an automated channel Technicon autoanalyzer (Seal Analytical continuous flow AutoAnalyzer AA3) using conventional colorimetric methods [62]. These measurements were performed in order to ensure that no deleterious effects, potentially associated with an eventual nutrient limitation or drastic variations of pH and/or oxygen level occurred during the time course of the incubation experiments.

### Effect of fresh leaves/thalli on dinoflagellate growth

Dinoflagellate cell densities were monitored at Days 0, 1, 3, 6, 8, and 10 by direct microscopic counts of cells. Maximum growth rates (μ_max_; expressed in day^-1^) were calculated according to Guillard [63] from the slope of a linear regression over the entire exponential phase of growth by the least squares fit of a straight line to the data after logarithmic transformation: μ_max_ = [Ln(N_t_)—Ln(N_0_)/(T_t_—T_0_)] where N_0_ and N_t_ are the cell densities (cells.mL^−1^) at the beginning (T_0_) and the end (T_t_) of the exponential phase, respectively. The EC_50_ (effective concentrations inducing a 50% reduction of dinoflagellates growth when compared to the control) were determined using curves that link the observed growth rates to the tested macrophyte weights (FW).

### Effect of fresh leaves/thalli on dinoflagellate photosynthetic activity

The efficiency of the photosynthetic apparatus of the four dinoflagellate species was assessed with a portable pulse amplitude modulated fluorometer AquaPen-C AP-C 100 device (Photon Systems Instruments, Czech Republic), measuring chlorophyll fluorescence parameters and by using the FluorPen 1.0.4.2 software to access the data. The OJIP protocol (Chlorophyll Fluorescence Induction Kinetics, [64]) was applied after a 30-min dark-adaptation period before measurements. Photosynthetic activity was monitored at Days 1, 3, 6, and 10 and the ratio Fv/Fm, corresponding to the maximum quantum yield of Photosystem II (PSII), was used to characterize the physiological status of the microalgae, as it is classically done [65].

### Effect of fresh leaves/thalli on dinoflagellate morphology

Qualitative observations of the dinoflagellate cell morphology were performed microscopically (at 400x magnification) at the end of each experiment (Day 10). Up to 30 cells of each culture (controls and treatments) were photographed and analyzed using an inverted microscope (Zeiss Axiovert 25) connected to a camera (Canon G3). For some experiments, cells were stained with 4’,6-diamidino-2-phenylindole dihydrochloride (DAPI) and nuclear DNA was observed (at 630x magnification) with a Zeiss microscope (Zeiss Axioimager Z1 upright microscope).

### Effect of fresh leaves/thalli on dinoflagellate toxin production

For experiments with *O*. cf. *ovata*, *P*. *lima*, and *A*. *pacificum*, toxin profiles and contents were analyzed in order to assess the effect of the tested macrophytes on the toxin production of each dinoflagellate. A defined volume of the cultures was harvested at Day 10. Cells were centrifuged at 3500 x g for 10 min at 4°C and the supernatant was carefully removed. The pellets were stored at -20°C until toxin analysis. For *O*. cf. *ovata* and *P*. *lima*, the methods used to analyze the toxins were those described in Ben Gharbia et al. [34]. Toxin analyzes were performed as described by Laabir et al. [42] for *A*. *pacificum*.

### Dinoflagellate behavior

During the time course of the experiments (Days 1, 3, 6 and 10), the adhesion of the dinoflagellate species to the macrophyte leaves/thalli (vicinity and, attachment) was monitored and observed using an inverted microscope (Zeiss Axiovert 25).

### Statistical analyzes

Data were analyzed using one-way analysis of variance (ANOVA) in order to check for the existence of significant differences between control and treatments (different macrophyte weights). Two-way ANOVA (in considering both macrophyte weights and dinoflagellate species) was performed in order to clarify the relative sensitivity of the different dinoflagellates to each macrophyte, when exposed to the same gradient of thalli/leaf weights. In both cases, Tukey post-hoc tests were performed to segregate groups of similar responses within the different series of results (growth rate, photosynthetic activity and toxin production). The significance level was set at p < 0.05. Statistical analyzes were performed using the software SigmaStat (v3.5, Systat Software Inc.).

## Results

### Experimental conditions

For all experiments, no significant differences (p > 0.05) were observed between the pH and dissolved oxygen values of the controls and the different treatments. Nutrient analyzes showed a decrease in NO_3_^-^ and PO_4_^3-^ concentrations throughout the duration of the incubation experiments (see S1 Appendix). Nutrient concentrations at the end of our experiments remained at saturated levels for dinoflagellate requirements. Mean initial and final concentrations ranged between 418.5–484.4 (Day 0) and 192.4–376.6 μmol.L^-1^ (Day 10) for NO_3_^-^ and between 16.8–18.0 (Day 0) and 9.1–10.1 μmol.L^-1^ (Day 10) for PO_4_^3-^. The minimum values for NO_3_^-^ and PO_4_^3-^ were recorded in the presence of *U*. *rigida* thalli and were equal to 177.8 μmol.L^-1^ and 8.0 μmol.L^-1^, respectively.

### Effects of fresh macrophyte leaves/thalli on dinoflagellate growth

**Fig 2:**
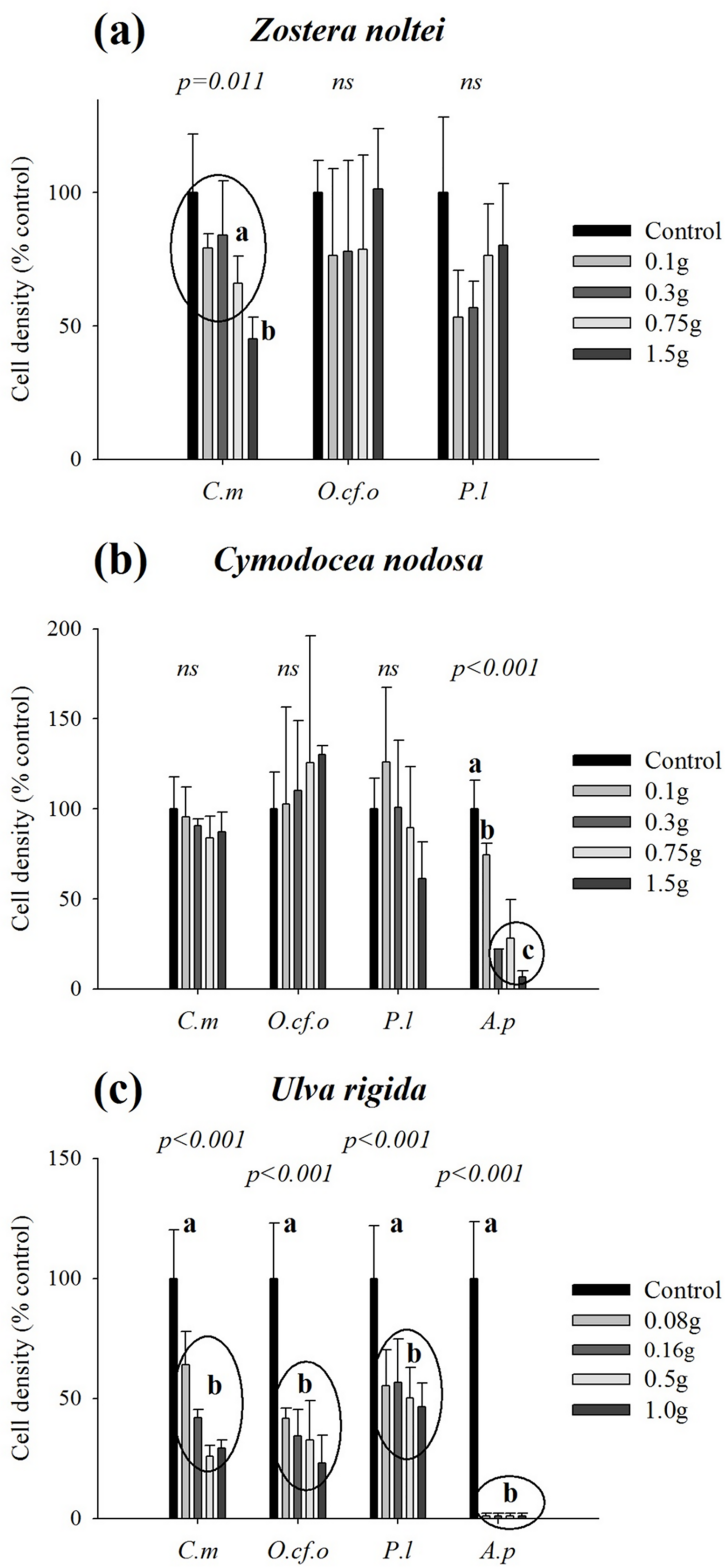
**Normalized final cell densities (% of the control) of the tested dinoflagellates, exposed to different weights of fresh leaves/thalli of *Zostera noltei* (a), *Cymodocea nodosa* (b) and *Ulva rigida* (c) at the end of the experiments (Day 10).** Error bars correspond to the standard deviation (N = 3 replicates). The inscription ‘ns’ above bars indicates a statistically non-significant one-way ANOVA. ‘p-values’ associated with significant one-way ANOVA are provided; in such cases and for each dinoflagellate species, values that did not differ at the 0.05 level (Tukey post-hoc test) are assigned the same letter. (*C*.*m*: *Coolia monotis*; *O*.cf.*o*: *Ostreopsis* cf. *ovata*; *P*.*l*: *Prorocentrum lima*; *A*.*p*: *Alexandrium pacificum*).

**Fig 3:**
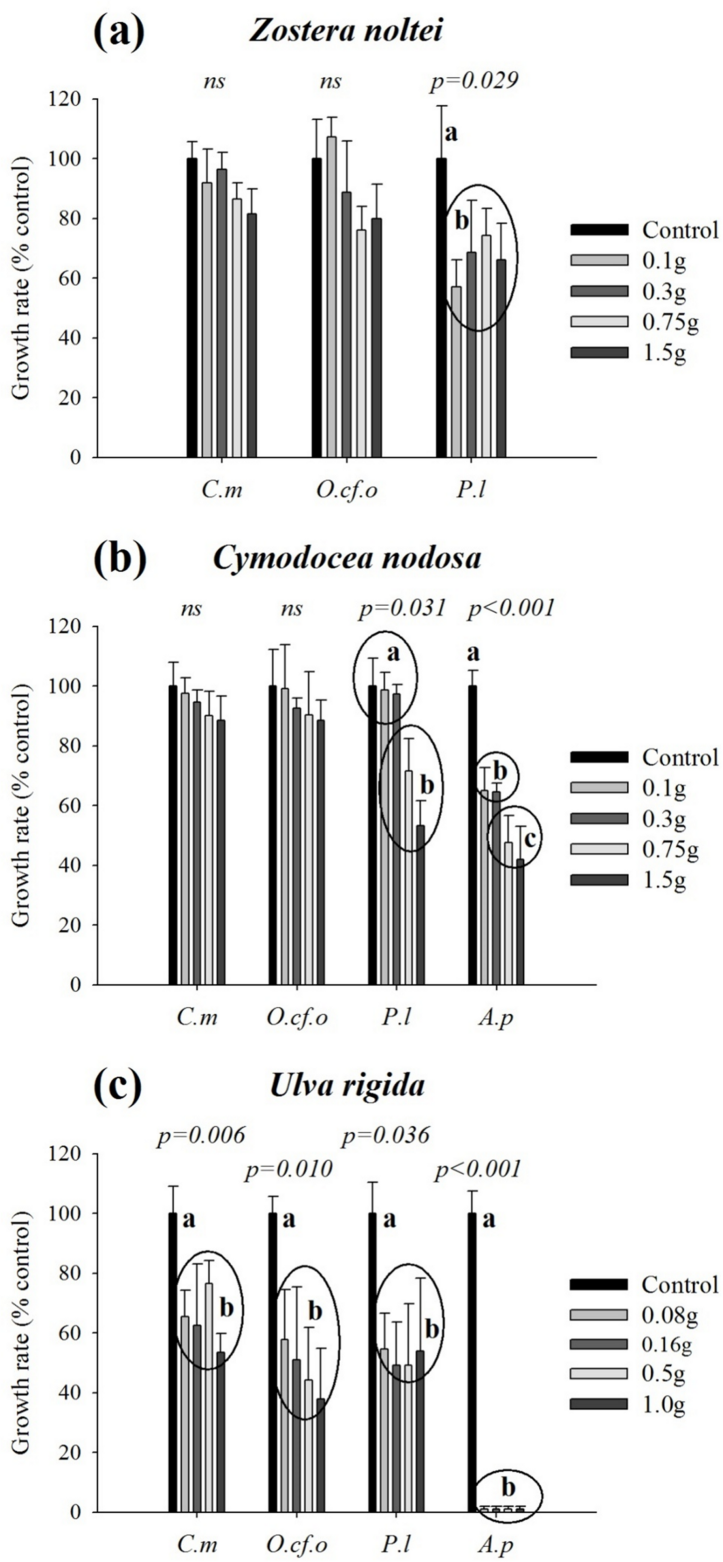
**Normalized maximum growth rates (% of the control) of dinoflagellate cells growing with fresh leaves/thalli of *Zostera noltei* (a), *Cymodocea nodosa* (b) and *Ulva rigida* (c).** Error bars correspond to the standard deviation (N = 3 replicates). The inscription ‘ns’ above bars indicates a statistically non-significant one-way ANOVA. ‘p-values’ associated with significant one-way ANOVA are provided; in such cases and for each dinoflagellate species, values that did not differ at the 0.05 level (Tukey post-hoc test) are assigned the same letter. (*C*.*m*: *Coolia monotis*; *O*.cf.*o*: *Ostreopsis* cf. *ovata*; *P*.*l*: *Prorocentrum lima*; *A*.*p*: *Alexandrium pacificum*).

*Zostera noltei* fresh leaves reduced the growth of the three benthic dinoflagellates. Compared to the controls (macrophyte free), cell density reduction at the end of the experiment (Day 10), was about 20–47% for *P*. *lima* and 21–24% for *O*. cf. *ovata* (across all treatments), but none of these apparent inhibitions was statistically significant (one-way ANOVA, F = 2.572, p = 0.103 and F = 0.581, p = 0.683, respectively; Fig 2A). In contrast, a pronounced inhibition was observed for *C*. *monotis* (one-way ANOVA, F = 5.813, p = 0.011), which was up to 55% for the highest weight tested. This treatment was statistically different from all the others, which were aggregated by the Tukey post-hoc procedure (Fig 2A). The effect of *Z*. *noltei* on growth rates was statistically significant only for *P*. *lima* (one-way ANOVA, F = 4.254; p = 0.029) with a decline ranging between 26% and 43%. Nevertheless, this decrease was independent from the weight of the tested macrophyte (Fig 3A). Growth rate reduction varied between 11% and 24% for *O*. cf. *ovata* and between 4% and 18% for *C*. *monotis*, but in both cases, no statistically significant differences were recorded (one-way ANOVA, F = 1.588, p = 0.252 and F = 2.912, p = 0.078, respectively; Fig 3A). Two-way ANOVA, performed on growth rates, confirmed that the effects of the dinoflagellate species and those of the different treaments were statistically significant (F = 8.201, p = 0.001 and F = 4.099, p = 0.009, for the effects of the dinoflagellate species and of the different treatments, respectively). Post-hoc tests failed to separate *C*. *monotis* and *O*. cf. *ovata*, but have discriminated *P*. *lima*, which suggests a higher sensitivity of this species to *Z*. *noltei* when compared to the two other benthic dinoflagellates.

In the presence of *C*. *nodosa* leaves, *O*. cf. *ovata*, *P*. *lima* and *C*. *monotis* cell densities were not statistically different from those of the controls at the end of the experiments (one-way ANOVA, F = 0.280, p = 0.884; F = 1.670, p = 0.233 and F = 0.728, p = 0.593 respectively; Fig 2B).

The growth rates of the three benthic dinoflagellates were affected differently. A slight inhibition, ranging between 1–12% for *O*. cf. *ovata* (one-way ANOVA: F = 0.262, p = 0.896) and between 3–11% for *C*. *monotis* (one-way ANOVA: F = 1.457, p = 0.286), was recorded (Fig 3B). A statistically significant negative effect was observed only on *P*. *lima* growth rates (one-way ANOVA: F = 4.138, p = 0.031), and two different clusters of treatments have been segregated by the Tukey post-hoc test with a decrease of about 30–40% for the two most concentrated treatments. In contrast, the planktonic *A*. *pacificum* was dramatically inhibited by the presence of *C*. *nodosa* leaves, which induced a strong decrease in cell densities (one-way ANOVA: F = 59.932, p < 0.001), up to 93% for the highest weight tested (Fig 2B). Highly significant growth-rate inhibitions (ranging between 35% and 58%) were observed (one-way ANOVA: F = 51.925, p < 0.001). The effect levels depended on the macrophyte weight as highlighted by the Tukey post-hoc test, which distinguished three groups of treatments (Fig 3B). The EC_50_ value for *A*. *pacificum* cultured with fresh *C*. *nodosa* leaves was 3.4 g.L^-1^ FW (equivalent to 0.72 g.L^-1^ DW). Two-way ANOVA, performed on growth rates, confirmed that the effects of the dinoflagellate species and those of the different treaments were statistically significant (F = 13.732, p < 0.001 and F = 21.900, p < 0.001, for both factors, respectively). Post-hoc tests highlighted the highest sensitivity of the planktonic dinoflagellate *A*. *pacificum*, which was systematically segregated regardless of the concentration tested. *P*. *lima* and *A*. *pacificum* were grouped in a cluster significantly different from *C*. *monotis* and *O*. cf. *ovata* for the two highest concentrations, indicating again a more pronounced sensitivity of *P*. *lima* when compared to the two other benthic species.

The macroalgae *U*. *rigida* induced the most important and significant decrease in cell abundances of the three benthic species after 10 days of co-cultures (p < 0.001, for the three dinoflagellates; Fig 2C). Compared to the controls, growth rates decreased between 42% and 62% for *O*. cf. *ovata* (one-way ANOVA: F = 6.002, p = 0.010), between 38% and 51% for *P*. *lima* (one-way ANOVA: F = 3.949, p = 0.036), and between 35% and 47% for *C*. *monotis* (one-way ANOVA: F = 7.055, p = 0.006) (Fig 3C). The EC_50_ values for *U*. *rigida* were 1 g.L^-1^ (equivalent to 0.153 g.L^-1^ DW) for *O*. cf. *ovata* and 2.35 g.L^-1^ (equivalent to 0.36 g.L^-1^ DW) for *P*. *lima*. Inhibition did not exceed 47% for *C*. *monotis* cells exposed to *U*. *rigida*, and the calculation of the EC_50_ value was thus not possible. The growth of *A*. *pacificum* was highly affected by *U*. *rigida* (one-way ANOVA: F = 529.376, p < 0.001): all of the *A*. *pacificum* cells exposed to the three highest weights tested died at Day 6, and the EC_50_ value was lower than 0.4 g.L^-1^ (equivalent to 0.06 g.L^-1^ DW) (Fig 3C). In all cases, Tukey post-hoc tests clearly separated the controls from the different treatments that were similar. Two-way ANOVA, performed on growth rates, confirmed that the effects of the dinoflagellate species and those of the different treaments were statistically significant (F = 41.918, p < 0.001 and F = 37.847, p < 0.001, for both factors, respectively). Tukey post-hoc tests clearly separated *A*. *pacificum* from the three benthic dinoflagellates, whose responses were not statistically different from each other. When a two-way ANOVA was performed without considering *A*. *pacificum*, no more additional effects of the dinoflagellate species were identified (F = 2.784, p = 0.078), which confirmed their similar responses when exposed to *U*. *rigida* thalli.

### Effect of fresh leaves/thalli on dinoflagellate photosynthetic activity

For all experiments, Fv/Fm values of controls increased systematically during the time course of the incubations, and were associated with healthy cultures (median values: 0.56, 0.60, 0.62, and 0.62 for Days 1, 3, 6, and 10, respectively, and in considering all the control replicates of the 11 co-incubation experiments).

**Fig 4:**
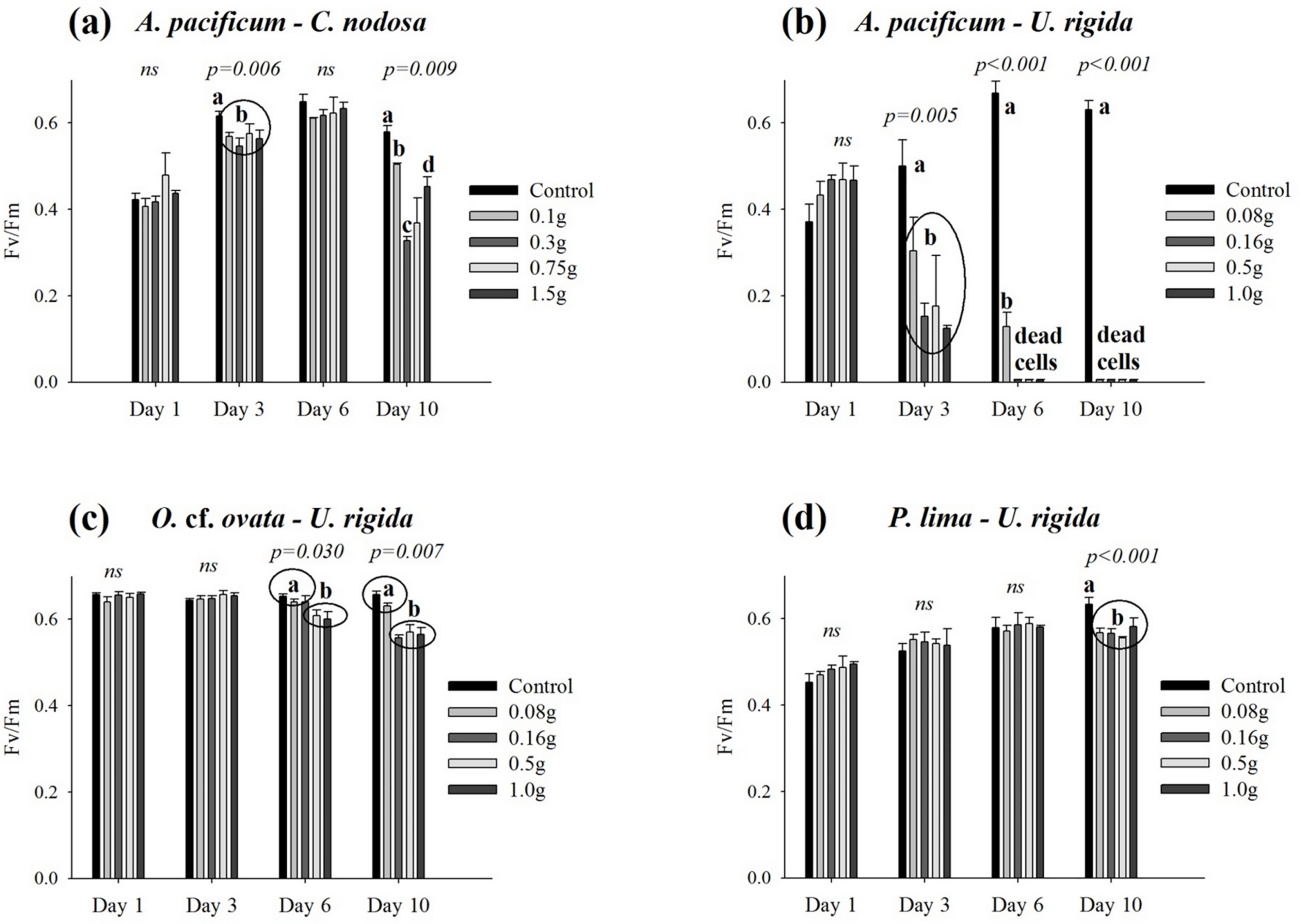
**Fv/Fm ratio (maximum quantum yield of Photosystem II) of *Alexandrium pacificum* cells in co-culture with fresh leaves/thalli of *Cymodocea nodosa* (a) and *Ulva rigida* (b); and of *Ostreopsis* cf. *ovata* (c) and *Prorocentrum lima* (d) cells growing with *Ulva rigida* thalli.** Error bars correspond to the standard deviation (N = 3 replicates). The inscription ‘ns’ above bars indicates a statistically non-significant one-way ANOVA. ‘p-values’ associated with significant one-way ANOVA are provided; in such cases and for each day, values that did not differ at the 0.05 level (Tukey post-hoc test) are assigned the same letter.

Results did not reveal any effects of fresh leaves from the two magnoliophytes *Z*. *noltei* and *C*. *nodosa* on the photosynthetic activity of the three benthic dinoflagellates. The maximum quantum yields of PSII (Fv/Fm ratio) remained consistently elevated, between 0.6 and 0.7, even for the highest macrophyte weights tested. In contrast, *C*. *nodosa* leaves induced a statistically significant decrease of the maximum quantum yield of PSII of *A*. *pacificum* cultures (one-way ANOVA performed on Day 3: F = 6.909, p = 0.006 and F = 6.139, p = 0.009 on Day 10), ranging between 13% and 44% at the end of the experiment (Day 10) (Fig 4A). This emphasizes again the higher sensitivity of the planktonic dinoflagellate in its physiological responses.

A dramatic effect was observed when *A*. *pacificum* was co-incubated with *U*. *rigida*. This macroalgae induced a strong inhibition of the photosynthetic efficiency after 3 days of exposure to the thalli (one-way ANOVA performed on Day 3: F = 7.302, p = 0.005) for all treatments (Fig 4B). At Day 6, the reduction of Fv/Fm values for the lowest weight tested reached 86% when compared to the control (one-way ANOVA performed on Day 6: F = 21.382, p < 0.001), and all cells were dead for the three other treatments. No measurements were performed at Day 10, because, except for the controls, all *Alexandrium* cells died. The effect of *U*. *rigida* on the photosynthetic activity of the three benthic species was clearly less important than that observed for *A*. *pacificum*. A statistically significant decrease of the Fv/Fm ratio was observed for *O*. cf. *ovata* at Day 6 (one-way ANOVA: F = 4.213, p = 0.030) for the two highest weights tested (0.5 g and 1.0 g FW). This decrease was not statistically different between the two treatments (Tukey post-hoc test). The inhibition seemed more pronounced at the end of the experiment (one-way ANOVA performed on Day 10: F = 6.731, p = 0.007) for the three highest treatments (0.16 g, 0.5 g and 1.0 g FW) with Fv/Fm decreases ranging between 13 and 15% (Fig 4C). The Tukey post-hoc test failed to separate these three treatments that have been clustered into a single group. Except for the treatment 0.16 g FW of *U*. *rigida* thalli, which was not significantly effective on Day 6 (clustered in the same group ‘a’ as the control) but was active on Day 10 (clustered in group ‘b’), the overlapping of error bars for the two more concentrated treatments (on Day 6 and Day 10) indicated that the observed inhibitions at the two dates were not statistically different. For *P*. *lima*, a statistically significant reduction of Fv/Fm (4%-12%) was recorded only on Day 10 (one-way ANOVA: F = 16.720, p < 0.001) (Fig 4D). No significant effect was observed for *C*. *monotis* cultures, with a marginal decrease of the Fv/Fm ratio on Day 10 that did not exceed 6% for the highest treatment when compared to the control (one-way ANOVA: F = 0.667, p = 0.629).

### Effect of the macrophytes on dinoflagellate cell morphology

**Fig 5:**
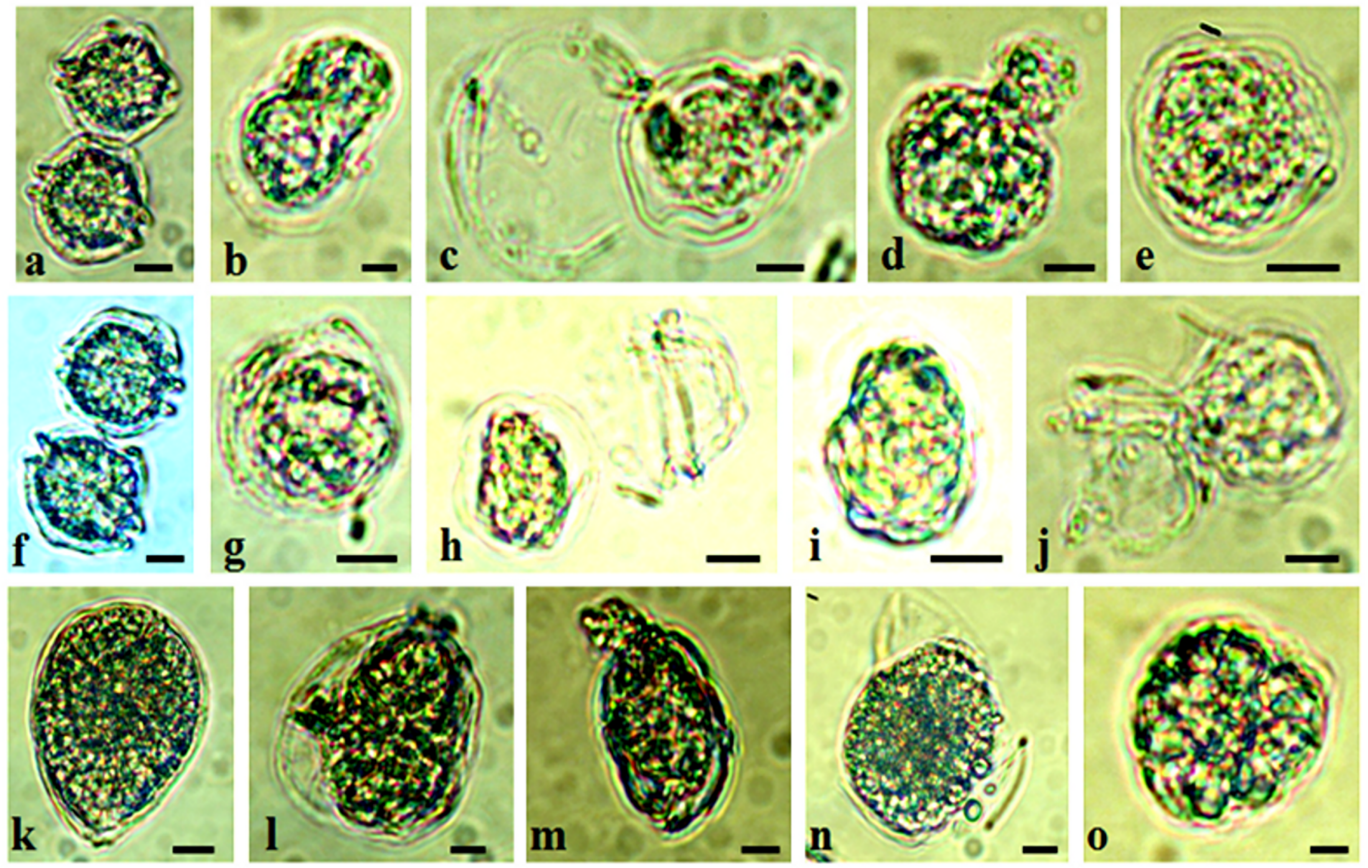
Light microscope observations of morphological damages of vegetative cells of the targeted dinoflagellate species. Photographs of *Alexandrium pacificum* cells cultured with *Cymodocea nodosa* (a-e) and *Ulva rigida* (f-j); and of *Ostreopsis* cf. *ovata* cultured with *Ulva rigida* (k-o). a,f,k = control cells; b-e, g-j, l-o = cells under increasing macrophyte weights. Scale bars, 10 μm.

Cell morphology observations at the end of the co-culture experiments did not reveal any major morphological damage on the cells of the three benthic dinoflagellates in the presence of *Z*. *noltei* and *C*. *nodosa*. In contrast, after 10 days of *A*. *pacificum* co-culture with *C*. *nodosa* (0.3, 0.75 and 1.5 g FW) deformed cells, membrane lysis and an important degradation of the intracellular contents were observed at a large scale (Fig 5A–5E).

In the presence of *U*. *rigida*, empty thecae or lysed and deformed *A*. *pacificum* cells were observed for all treatments at the end of the experiment (Fig 5F–5J). *O*. cf. *ovata* cells co-cultured with *U*. *rigida* thalli exhibited structural damage and aberrant forms (lysed or small-rounded cells) (Fig 5K–5O) while *P*. *lima* and *C*. *monotis* cells were not impacted.

**Fig 6:**
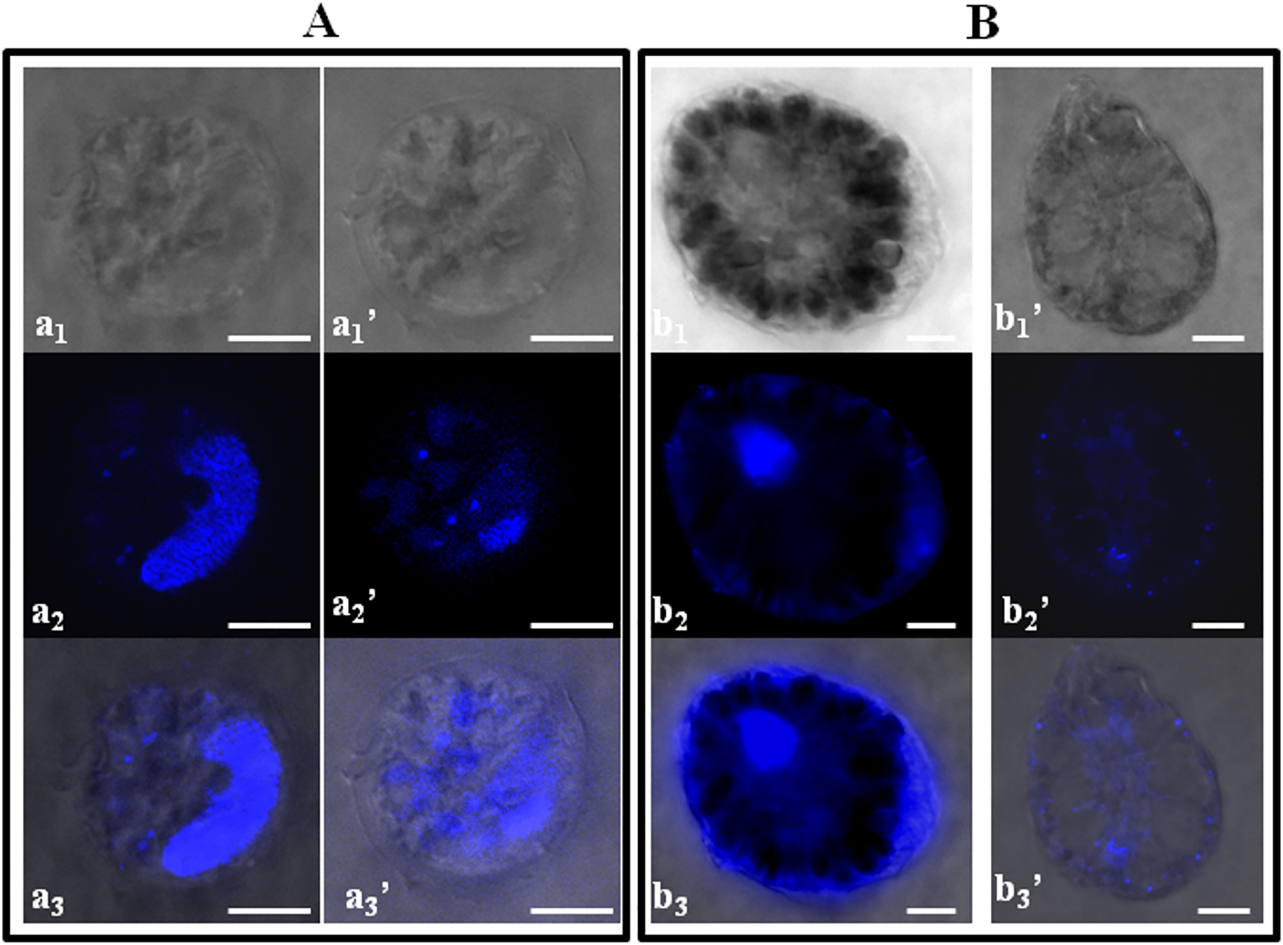
Light (a_1_,a_1_’,b_1_,b_1_’), epifluorescence (a_2_,a_2_’,b_2_,b_2_’) and superposed light-epifluorescence (a_3_,a_3_’,b_3_,b_3_’) microscope photographs of dinoflagellate vegetative cells cultured with *Ulva rigida* thalli. **A**: *Alexandrium pacificum* cells (a_1_-a_2_-a_3 =_ control, a_1_’-a_2_’-a_3_’ _=_ cell exposed to 0.16g (FW) of *Ulva rigida* after 3 days of co-culture). **B**: *Ostreopsis* cf. *ovata* cells (b_1_-b_2_-b_3 =_ control; b_1_’-b_2_’-b_3_’ _=_ cell exposed to 1g FW of *Ulva rigida* after 10 days of co-culture). Scale bars, 10 μm.

Dinoflagellate vegetative cells exposed to *U*. *rigida* thalli were also observed under fluorescent light after staining the nucleus with DAPI, and we noticed scattered and irregular DNA for *A*. *pacificum* and *O*. cf. *ovata* (Fig 6A and 6B). No effects were found on *P*. *lima* and *C*. *monotis* cells, which were characterized by a regularly shaped nucleus and condensed chromosomes despite the treatment.

### Effect of fresh leaves/thalli on dinoflagellate toxin production

**Fig 7:**
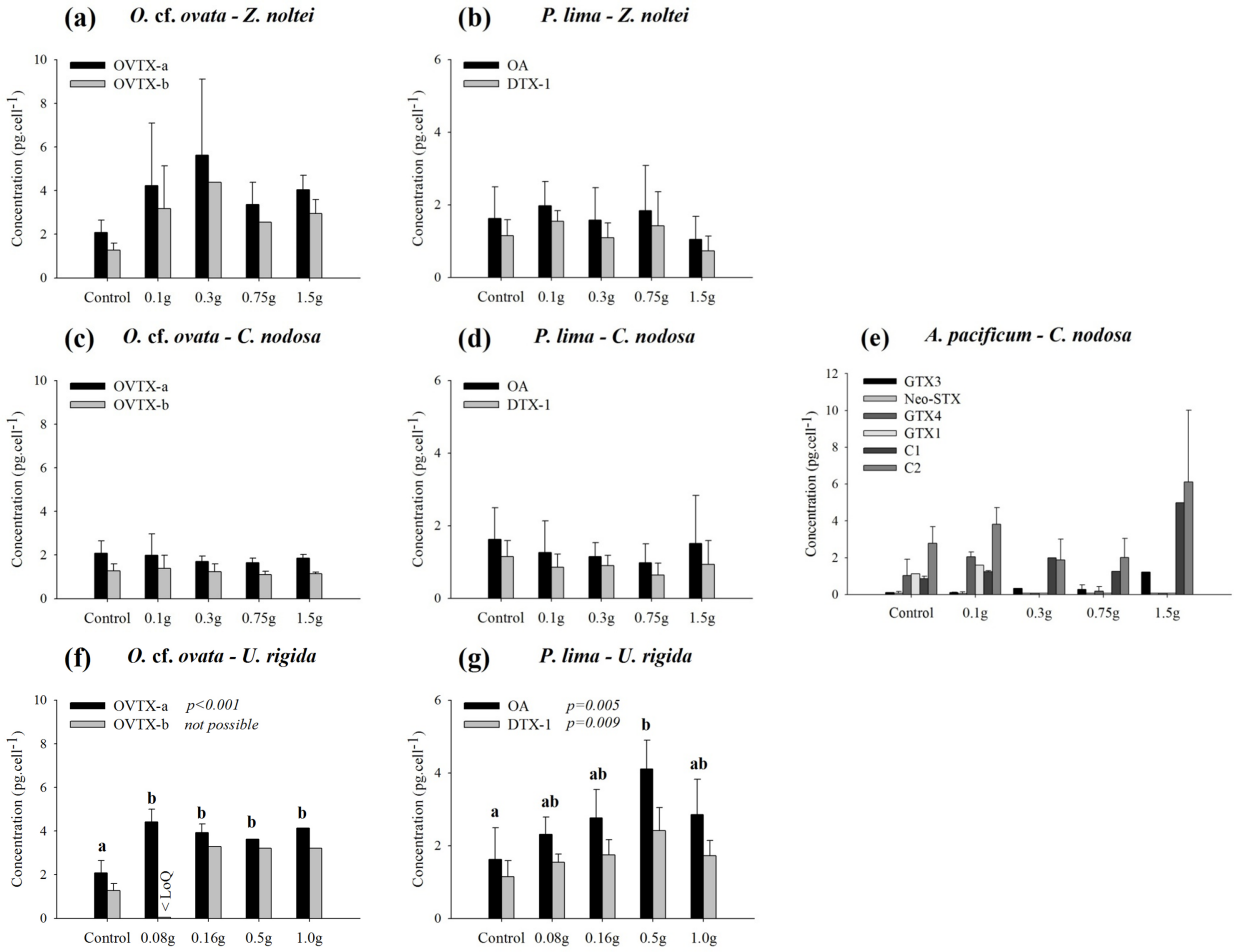
Cellular toxin contents (pg.cell^-1^) at the end of the experiments (after 10 days) of *Ostreopsis* cf. *ovata* (*O*. cf. *ovata*) and *Prorocentrum lima* (*P*. *lima*) in presence of the leaves/thalli of *Cymodocea nodosa* (*C*. *nodosa*), *Zostera noltei* (*Z*. *noltei*) and *Ulva rigida* (*U*. *rigida*), and of *Alexandrium pacificum* (*A*. *pacificum*) in presence of *C*. *nodosa* leaves. OVTX-a: Ovatoxin-a; OVTX-b: Ovatoxin-b; OA: Okadaic Acid; DTX-1: Dinophysistoxin-1; Neo-STX, GTX1, GTX3 and GTX4: Carbamoyl toxins; C1 and C2: N-sulfocarbamoyl toxins. ‘< LoD’ and ‘< LoQ’ indicate ‘< Limit of Detection’ and ‘< Limit of Quantification’, respectively. Error bars correspond to the standard deviation (N = 3 replicates, except for control (*O*. cf. *ovata* and *P*. *lima*) for which the controls of the three experiments have been pooled, N varying between 3 and 9 depending on the considered toxin). When only one among the three triplicates of each treatment was above LoD or LoQ, standard deviation was not calculable, and there is thus no error bar in such cases.

Toxin contents measured in dinoflagellate cells at the end of the different co-culture experiments revealed contrasting patterns. The exposure of *O*. cf. *ovata* cells to *Z*. *noltei* leaves seemed to induce a stimulation of ovatoxin production (OVTX-a and OVTX-b). However, results were not statistically significant due to the important within-treatments variability (Fig 7A; one-way ANOVA performed at Day 10: F = 2.124, p = 0.140 for OVTX-a, and F = 1.874, p = 0.247 for OVTX-b). For *P*. *lima*, the observed concentrations of okadaic acid (OA) and of dinophysistoxin-1 (DTX-1) after ten days of co-incubation with *Z*. *noltei* leaves were not different from those measured in the controls (Fig 7B; one-way ANOVA: F = 0.481, p = 0.750 for OA, and F = 1.165, p = 0.363 for DTX-1).

The toxin content of *O*. cf. *ovata* cells exposed to *C*. *nodosa* leaves (Fig 7C) was not statistically different from that of the controls after ten days of co-incubations despite the treatment (one-way ANOVA performed at Day 10: F = 0.421, p = 0.791 for OVTX-a, and F = 0.259, p = 0.897 for OVTX-b). This was also the case for *P*. *lima* (Fig 7D; one-way ANOVA performed at Day 10: F = 0.439, p = 0.779 for OA, and F = 0.889, p = 0.493 for DTX-1). For *A*. *pacificum* (Fig 7E), the apparent increase of the cellular toxin contents was once again not statistically significant (one-way ANOVA performed at Day 10: F = 5.669, p = 0.096; F = 0.747, p = 0.479; F = 15.213, p = 0.060; and F = 1.566, p = 0.314 for GTX4, GTX3, C1 and C2 respectively). Some measurements were below the limit of detection (GTX1) or below the limit of quantification (Neo-STX), and it was not possible in such cases to perform one-way ANOVA.

In the presence of *U*. *rigida*, ovatoxin-a production by the *O*. cf. *ovata* strain was significantly enhanced (Fig 7F; one-way ANOVA performed at Day 10: F = 17.280, p < 0.001). Tukey post-hoc test clearly separated the controls from the different treatments. Several ovatoxin-b analyzes were below the limits of quantification (Fig 7F), and it was not possible to formally assess the potential impact of the macrophyte thalli. The stimulating effect of *U*. *rigida* on the toxin content of *P*. *lima* cells was also statistically confirmed (Fig 7G; one-way ANOVA performed at Day 10: F = 5.657, p = 0.005 for OA, and F = 4.969, p = 0.009 for DTX-1). Tukey post-hoc test clearly separated the control from the third treatment (0.5g), while all the other treatments corresponded to intermediary responses. Quantification of *A*. *pacificum* toxin content has not been performed: all cells died before the end of the experiment.

### Dinoflagellate behavior

Observations of dinoflagellate behavior in co-cultures revealed that cells of the three benthic strains covered the bottom of the flasks or were suspended in water and embedded in mucus rather than attached to the macrophyte leaves/thalli. For the planktonic *A*. *pacificum*, we did not observe any cell attachment to the macrophytes. In the presence of *Z*. *noltei* and *C*. *nodosa*, *O*. cf. *ovata* and *C*. *monotis* cells were observed on the leaf edges but not on the surface. They formed aggregates around the ends/extremities of the leaf and used it as a support to form mucus that encompassed the cells. *P*. *lima* cells colonized both the edges and the entire surface of *Z*. *noltei* and *C*. *nodosa* leaves. The same pattern was observed for *P*. *lima* co-cultured with *U*. *rigida*, even if the adhesion of the cells to the thalli was less important in comparison to the two other magnoliophytes. For *O*. cf. *ovata*, cells adhered mainly to the edges of *U*. *rigida* thalli, but were also in contact with the whole surface. We noticed that *O*. cf. *ovata* cell attachment was less important when the weights of *Ulva* thalli increased. As for *C*. *monotis*, cells did not cling a lot to the thalli, and were observed only on the edges.

Thus, direct contact between the dinoflagellate cells and the entire surface of the leaves/thalli (not only the edges) was observed solely for *P*. *lima* co-cultured with the three tested macrophytes and for *O*. cf. *ovata* co-cultured with *U*. *rigida* (See S2 Appendix).

## Discussion

In aquatic ecosystems, primary producers (microalgae and macroalgae) are known to compete for light and nutrients [66]. Macrophytes can make an environment unsuitable for microalgal growth by reducing light and O_2_ levels, increasing pH, uptaking nutrients, and releasing various allelopathic substances. In our study, co-cultures were conducted under stable environmental conditions. Normal pH and oxygen levels were recorded during the incubation period, and the residual concentrations of NO_3_^-^ and PO_4_^3-^ measured at the end of our experiments for both controls and treatments were above the limiting levels [67–69]. Thus, our results suggested that the observed inhibitory effects were mainly due to potential algicidal allelopathic compounds that could be released by the tested macrophytes, and that a shortage in nutrients or unsuitable pH or O_2_ levels could not be incriminated.

### Effect on growth and cell morphology

To our knowledge, studies characterizing the allelopathic effect of macrophytes on benthic dinoflagellates are rather rare. Table 1 summarizes the current knowledge of the allelopathic effects associated with *Ulva* spp. and *Zostera* spp. on the growth of HAB-forming dinoflagellate species investigated in various marine ecosystems.

**Table 1:** Reported allelopathic effects of *Ulva* spp. and *Zostera* spp. on harmful algal blooms dinoflagellate species in various marine ecosystems.

Macrophyte species and origin	Target dinoflagellate species and strains	Effects	Tested concentrations	Time course experiments	References
***Ulva* spp.**					
*Ulva rigida* (Conero Riviera, Italy)	*Ostreopsis* cf. *ovata* (OoAPn0807/E)	FT: GI (CR = 94%)	FT:1g.500mL^-1^ FW	20 Days	[26]
		FTF: GS	FTF: 24 g.L^-1^ FW	23 Days	
		DP: GI (CR = 17–37%), vegetative cells replaced by cysts	DP: 0.4–0.8–1.6 g.L^-1^ DW	18 Days	
*Ulva lactuca* (Old Fort Pond, Long Island, NY, USA)	*Prorocentrum minimum* (CCMP696)	FT: (CR = 24%_72h-725mg.L-1 DW_), DPAE: GI_180-360mg.L-1 DW_	FT ≈ 45-180-400-725 mg. L^-1^DW	5 Days	[54]
	*Karlodinium veneficum* (FR-6)	FT: (CR = 38%_72h-725mg.L-1 DW_), DPAE: GI_360mg.L-1DW_ + cells lysed after 5 days	DPAE ≈ 36-180-360-1800 mg. L^-1^DW	5 Days	
	*Karenia brevis* (CCMP2228)	FT: (CR = 50%_72h-725mg. L-1DW_), DPAE: GI_1800mg.L-1DW_ + cells lysed after 5 days			
	*Cochlodinium polykrikoides* (CP1)	FT: (CR = 29%_120h-400mg.L-1DW_), DPAE: **			
*Ulva fasciata*, *Ulva pertusa*, *Ulva arasakii*, *Ulva conglobota* (Nagasaki, Japan)	*Alexandrium catenella* (NIES-677)	PM: GI (HDTA-ALA:CR<30%, ODTA: 30<CR<69%_25μg.mL-1_ / CR<30%_5μg.mL-1_)	PM (HDTA-ALA-ODTA): 5–25 μg.mL^-1^	4 Hours	[53]
	*Cochlodinium polykrikoides* (ND-14)	PM: GI (HDTA-ALA-ODTA:CR<30%)			
	*Karenia mikimotoi* (NIES-680)	PM: GI (HDTA-ALA-ODTA:CR>70%)			
	*Heterocapsa circularisquama* (ND-12)	PM: GI (HDTA-ALA-ODTA:30<CR<69%)			
	*Heterocapsa triquetra* (NIES-7)	PM: GI (HDTA-ALA: 30<CR<69%_25μg.mL-1_/CR<30%_5μg.mL-1_, ODTA: CR>70%_25μg.mL-1_/ 30<CR<69%_5μg.mL-1_)			
	*Scrippsiella sweeneyae* (NIES-684)	PM: GI (HDTA-ALA: 30<CR<69%_25μg.mL-1_/CR<30%_5μg.mL-1_, ODTA: CR>70%_25μg.mL-1_/ 30<CR<69%_5μg.mL-1_)			
	*Prorocentrum minimum* (ND-34)	PM: GI (HDTA-ALA-ODTA: 30<CR<69%_25μg.mL-1_ / CR<30%_5μg.mL-1_)			
	*Prorocentrum sigmoides* (NIES-683)	PM: GI (HDTA-ALA-ODTA: 30<CR<69%_25μg.mL-1_ / CR<30%_5μg.mL-1_)			
	*Scrippsiella trochoidea* (NIES-369)	PM: GI (HDTA-ALA-ODTA:CR>70%_25μg.mL-1_ / 30<CR<69%_5μg.mL-1_)			
*Ulva lactuca* (Nanao island, South China Sea)	*Alexandrium tamarense* (**)	FT: GI (CR = 48%)	FT: 0.8 g.L^-1^FW	12 Days	[112]
		FTF: GI first 2 days (recovered in the following days)	FTF: 80 g.L^-1^FW	**	
		DP: GI (EC_50_ = 0.19 g.L^-1^ DW)	DP ≈ 0.5-1-2 g.L^-1^DW	3 Days	
*Ulva fasciata* (**)	*Alexandrium tamarense* (**)	PM: GI (α-linolenic acid: LC_50_ = 66.06; linoleic acid: LC_50_ = 98.40 μg.mL^-1^)	PM: α-linolenic acid and linoleic acid	24 Hours	[113]
	*Alexandrium taylori* (**)	PM: GI (α-linolenic acid: LC_50_ = 35.30; linoleic acid: LC_50_ = 72.47μg.mL^-1^)	≈10^−2^–10^−1^–10^0^–10^1^−10^2^−10^3^ μg.mL^-1^		
	*Gymnodinium impudicum* (**)	PM: GI (α-linolenic acid and linoleic acid: LC_50_ > 1,000 μg.mL^-1^)			
	*Heterocapsa circularisquama* (**)	PM: GI (α-linolenic acid and linoleic acid: LC_50_ > 1,000 μg.mL^-1^)			
*Ulva linza* (Taiping Angle of Qingdao, China)	*Prorocentrum donghaiense* (**)	FT: GI (EC_50_ = 0.4 g.L^-1^DW)	FT: 0.625–1.25–2.5-5-10 g.L^-1^FW	10 Days	[114]
		FTF: GI (CR≈94%)	FTF: 80 g.L^-1^FW	≈10 Days	
		DP: GI (EC_50_ = 0.1 g.L^-1^DW)	DP ≈ 0.15–0.3–0.6–1.2–2.4 g.L^-1^DW	10 Days	
		AE: GI (EC_50_ = 1.5 ppt)	AE ≈ 0.1–0.2–0.4–0.8–1.6 ppt	5 Days	
		ME: GI (EC_50_ = 0.02 ppt)	ME≈ 0.025–0.05–0.1–0.2–0.4 ppt	5 Days	
*Ulva pertusa* (Huiquan Bay, China)	*Prorocentrum donghaiense* (**)	FT: GI (LT_50_ = 37.9 h), FTF: GI (≈5%)	FT: 3.4 g.L^-1^FW	≈216 Hours	[115]
	*Alexandrium tamarense* (**)	FT: GI (LT_50_ = 59.8 h), FTF: GI (≈27%)	FTF: 3.4 g.L^-1^FW	≈216 Hours	
	*Scrippsiella trochoide* (**)	FT: GI (LT_50_ = 63.6 h), FTF: GI (≈7%)			
	*Amphidinium carterae* (**)	FT: GI (≈34%), FTF: GS (≈78%)			
*Ulva pertusa* ^**a**^, *Ulva linza* ^**b**^ (Coast of Qingdao, China)	*Prorocentrum micans* (**)	FT: GI (EC_50_ = 1.8^**a**^ - 2.3^**b**^ g.L^-1^FW)	FT ≈ 0.625–1.25–2.5-5-10 g.L^-1^FW	10 Days	[70]
		FTF: No significant inhibitory effects	FTF: 40 g.L^-1^FW	10 Days	
		DP: GI (EC_50_ = 0.7 ^**a**^**—**0.8 ^**b**^ g.L^-1^DW)	DP ≈ 0.15–0.3–0.6–1.2–2.4 g.L^-1^DW	10 Days	
		AE: GI (EC_50_ = 0.7 ^**a**^**—**1.0 ^**b**^ ppt)	AE: 0.1 to 1.6 ppt	6 Days	
		ME: GI (EC_50_ = 0.015 ^**a**^**—**0.017 ^**b**^ ppt)	ME: 0.025 to 0.4 ppt	6 Days	
*Ulva conglobota* ^**a**^, *Ulva fasciata* ^**b**^, *Ulva pertusa* ^**c**^ (Nagasaki Beach, Japan)	*Gymnodinium mikimotoi* (NIES-680)	ME: GI (Mortality = 10.0% ^**a**^—71.3% ^**b**^- 10.1% ^**c**^)	ME:** (10μL of extract.mL^-1^)	4 Hours	[52]
*Ulva pertusa* (**)	*Alexandrium tamarense* (**)	FT: GI (≈70%)	FT: 12.5 g.L^-1^FW	≈11 Days	[116]
		FTF: No significant inhibitory effects	FTF: 80 g.L^-1^FW	≈7 Days	
*Ulva pertusa*: Non-sexual ^**a**^ (**) Sexual ^**b**^ strain (Coast of Qingdao, China)	*Alexandrium tamarense* (**)	FT: GI (EC_50_ = 2 ^**a**^—2.5 ^**b**^ g.L^-1^FW)	FT: 0.625–1.25–2.5-5-10 g.L^-1^FW	10 Days	[51]
		FTF: Slight, not significant GS	FTF: 100 g.L^-1^FW	10 Days	
		DP: GI (EC_50_ = 0.6 ^**a**^**—**0.8 ^**b**^ g.L^-1^DW)	DP: 0.15–0.3–0.6–1.2–2.4 g.L^-1^DW	10 Days	
***Zostera* spp.**					
*Zostera noltii* ^**a**^, *Zostera marina* ^**b**^ (Thau lagoon and Arcachon bay, France)	*Alexandrium catenella* (ACT03)	AE: GI (EC_50_ = 0.76 ^**a**^—0.82 ^**b**^ g.L^-1^DW)	AE ≈ 0.13 to 3.34g of extract.L^-1^	72 Hours	[29]
		ME: GI (EC_50_ = 0.12 to 0.42 ^**a**^—0.09 to 0.29 ^**b**^ g.L^-1^DW)	ME ≈ 0.065 to 2g of extract.L^-1^	72 Hours	
		AE+ME: Loss of motility / Loss of thecae / Retracted intracellular contents / Degradation in intracellular organelles / Some cells stopped their division / Scattered and irregular DNA.			
*Zostera marina* (Roberts Bank, Canada)	*Gonyaulax polyedra* (**)	ME: GI (CR = 100%: No viable cells after 30 Days). Reduced swimming speed / Loss of motility / Loss of thecae / Extruded protoplasts / Cells disintegration.	ME(black leaves): 1.1 mg.mL^-1^DW	30 Days	[45]
	*Protogonyaulax tamarensis* (**)				

**FT** = Fresh Tissues, **FTF** = Fresh Tissue Filtrate (initial dose addition), **DP** = Dry Powder, **DPAE** = Dry Powder Aqueous Extracts, **PM** = Pure Molecules, **AE** = Aqueous Extracts, **ME** = Methanol Extracts.

**GI** = Growth Inhibition, **CR** = Cell Density Reduction, **GS** = Growth Stimulation, **FW** = Fresh Weight, **DW** = Dry Weight, **≈** = from graphs/tables

** = No Data.

**EC**_**50**_ = Effective Concentration inducing 50% reduction of dinoflagellate growth, **LC**_**50**_ = 50% Lethal Concentration, **LT**_**50**_ = Time at which 50% of the dinoflagellate cells are dead.

**HDTA-ALA-ODTA**: Hexadeca-4,7,10,13-tetraenoic acid (**HDTA**), α-linolenic acid (**ALA**), Octadeca-6,9,12,15-tetraenoic acid (**ODTA**).

(Macrophyte and Dinoflagellate species are named as cited in the references).

The present study highlighted contrasting effects of the three tested macrophytes on the growth of the four targeted dinoflagellate species. *U*. *rigida* exerted the highest algicidal effect, and the planktonic *A*. *pacificum* was the most sensitive dinoflagellate. Our results are in agreement with the observations of Accoroni et al. [26] who reported a significant allelopathic inhibitory effect of *U*. *rigida* thalli on *O*. cf. *ovata* (OoAPn0807/E). Alamsjah et al. [53] tested the effect of three algicidal compounds extracted from *Ulva* thalli on several planktonic HAB species, including *A*. *catenella* (NIES-677), and found a reduction of the growth of this dinoflagellate. Other authors showed that *Ulva* species could suppress the growth of different harmful dinoflagellates (Table 1). Here, for *U*. *rigida*, the EC_50_ values were 1 g.L^-1^ FW for *O*. cf. *ovata*, 2.35 g.L^-1^ FW for *P*. *lima*, and less than 0.4 g.L^-1^ FW for *A*. *pacificum*. Close EC_50_ values were reported for the effects of *Ulva pertusa* (1.8 g.L^-1^ FW) and *Ulva linza* (2.3 g.L^-1^ FW) species on the growth of *Prorocentrum micans* [70]. In contrast, it has been shown that *Ulva pertusa* may inhibit *Alexandrium tamarense* with an EC_50_ ranging between 2 and 2.5 g.L^-1^ FW [51], which is much higher than that found for the *U*. *rigida/A*. *pacificum* pair investigated in our experiments. This suggests that the allelopathic effect is highly species-specific.

Our results demonstrated that the growth of *A*. *pacificum* was highly affected in comparison to the three benthic species, which highlights an increased sensitivity of the physiological processes of this planktonic dinoflagellate to potential allelochemicals produced by the macrophytes. Benthic strains seem more resistant to substances that could be released by macrophytes; this could be explained by their permanent vicinity to the leaves/thalli, since they grow attached to the plant material. Hilt [71] supported our finding and highlighted a lower sensitivity of epiphyte species to allelochemicals. This author has reported in particular that epiphytic algae and cyanobacteria would be less vulnerable than planktonic species to the allelopathic effect of *Myriophyllum spicatum* and she has suggested that these organisms might have developed resistance against allelopathic substances released by macrophytes by a co-evolutionary process [7,72].

*Z*. *noltei* induced a moderate growth inhibition with statistically significant effects exerted only on *P*. *lima* whereas *O*. cf. *ovata* and *C*. *monotis* were not significantly affected. The potential effects of *Z*. *noltei* fresh leaves were not tested on *A*. *pacificum* in our study. However, Laabir et al. [29] have shown that *Z*. *noltei* and *Z*. *marina* crude extracts induced a strong inhibitory effect on *A*. *catenella* cells (ACT03 strain) even at very low concentrations (0.09 g of extract.L^-1^). De Wit et al. [73] have observed a delay in phytoplankton growth in the presence of *Z*. *noltei in situ* and hypothesized a direct interference related to the excretion or leaching of allelopathic substances by this macrophyte. Harisson and Chan [45], Harisson [47], and Harisson and Durance [46] have also suggested that chemicals released by *Zostera* leaves might reduce the growth of epiphytic microorganisms and decrease carbon uptake rates in diatoms.

In our study, *C*. *nodosa* induced the weakest inhibition on dinoflagellate growth in comparison with the other tested macrophytes. *C*. *nodosa* was not significantly efficient against *O*. cf. *ovata* and *C*. *monotis* (p > 0.05), moderately efficient against *P*. *lima* (p = 0.031) and significantly active against *A*. *pacificum* (p < 0.001). To our knowledge, there are no data in the literature about the allelopathic activity of *C*. *nodosa* leaves on dinoflagellates. However, some studies have examined the biological activity of this species. Kontiza et al. [48] have reported an antiproliferative effect of two biphenyl compounds isolated from *C*. *nodosa* on two lung cancer cell lines. Kontiza et al. [49] have also demonstrated an antibacterial activity of metabolites isolated from *C*. *nodosa* against multidrug-resistant and methicillin-resistant strains of *Staphylococcus aureus* as well as rapidly growing mycobacteria.

Our results showed that severe structural anomalies were induced by *C*. *nodosa* on *A*. *pacificum* cells. Similar effects were caused by *U*. *rigida* thalli that altered the cellular morphology of *A*. *pacificum* and *O*. cf. *ovata*. Previous studies have reported important degradations in intracellular contents, membrane disruption, and cell shrinkage of microalgal cells exposed to the allelopathic compounds of macrophytes [29,45,74]. Potential allelochemicals also seem to have genotoxic properties, as a DNA damage was observed for *O*. cf. *ovata* and *A*. *pacificum* cells co-cultured with *U*. *rigida*. DNA fragmentation and chromatin dispersion have been previously observed for *O*. cf. *ovata* cells when exposed to aldehydes from diatoms [75] and for *A*. *catenella* cells when exposed to *Zostera* extracts [29]. In our study, alterations in cell structures are in agreement with the observed high mortality rates associated with *U*. *rigida* thalli.

### Effect on photosynthesis

At the end of the experiment (Day 10), the photosynthetic efficiency of the three benthic species was not altered by *Z*. *noltei* and *C*. *nodosa* fresh leaves. Only *U*. *rigida* thalli induced a moderate decrease in Fv/Fm values of *O*. cf. *ovata* and *P*. *lima*. In contrast, the photosynthetic activity of the planktonic *A*. *pacificum* was strongly reduced by *C*. *nodosa* (up to 40% after 10 days in some cases) and by *U*. *rigida* with an important inhibition of the Fv/Fm ratio (up to 86% at Day 6 at the lowest weight tested).

Inhibition of the photosynthetic process by allelochemicals is a well known phenomenon in aquatic ecosystems, with the Photosystem II (PSII) being the main target [2,76]. Analysis of chlorophyll a fluorescence transient is a useful tool to assess several biophysical parameters related to the efficiency of PSII (fluxes of photons, excitons, electrons, and further metabolic events; [77]). Ye et al. [77] have found that the main photosynthetic inhibition targets by the macroalgae *Gracilaria lemaneiformis* on the dinoflagellate *Scrippsiella trochoidea* were a decrease in the quantity and size of antenna chlorophyll, in the number of active reaction centers, and in the photochemical efficiency of PSII, in addition to the blocking of the electron transport chain and the damage to the oxygen-evolving complex. Ye and Zhang [78] have also shown that dried thalli of *Gracilaria tenuistipitata* inhibited the photosynthesis of *P*. *micans*. They have attributed this effect to the decrease or the alteration of relevant parameters such as Fv/Fm, density of reaction centers, and electron transport per PSII cross-sections (RC/CS_0_ and ET_0_/CS_0_ when using OJIP terminology).

In freshwater ecosystems, different studies focused on the allelopathic effects of the macrophyte *Myriophyllum spicatum* on the photosynthetic activity of various microorganisms. Zhu et al. [25] have reported that allelochemicals isolated from this macrophyte were key agents in inhibiting the PSII and the whole chain activities of *Microcystis aeruginosa*. Purified tellimagrandin II and lipophilic extracts from *M*. *spicatum* were also found to inhibit the PSII of the cyanobacterium *Anabaena* sp. by the interruption of the photosynthetic electron transport between the primary and the secondary quinone electron acceptors (Q_A_ and Q_B_). These compounds were found to inhibit electron transport between Q_A_ and Q_B_ due to interference with non-heme iron [76]. It has been also reported that the cyclic sulfur compounds dithiolane and trithiane from *Chara globularis* can affect carbon uptake of diatoms and other phytoplankton species [79]. Nevertheless, the impairment of the photosynthetic activity of the targeted organisms by allelochemicals remains unclear, and the detailed mechanisms of action need further investigation.

### Effect on toxin production

To our knowledge, no data are available in the literature concerning the allelopathic effect of macrophytes on the toxin production of dinoflagellates. Our results revealed contrasting patterns. The observed increase in toxin contents of *O*. cf. *ovata* cells exposed to *Z*. *noltei* and *A*. *pacificum* cells exposed to *C*. *nodosa* leaves was not statistically confirmed due to the important within-treatment variability. Only, *U*. *rigida* thalli induced a significant stimulation of the toxin production of the two benthic dinoflagellates *O*. cf. *ovata* and *P*. *lima*. The induced effects were not dose-dependent for *O*. cf. *ovata*, as it seemed to be the case for *P*. *lima*. We can hypothesize that microalgae exposed to stressful conditions first enhance their toxin production, then, when the cell metabolism is altered and/or structural damages appear, the microorganisms may reduce or lose their capacity to produce toxins. Factors affecting the toxin production remain poorly known and results are often contradictory [80]. An enhancement of toxicity levels in P-limited dinoflagellate cultures and low toxin contents in N-limited cultures have been reported [80–82]. But, Vanucci et al. [83] found a significant increase in okadaic acid amounts of *P*. *lima* cells under both N and P limitations, while Vanucci et al. [84] observed a decrease in toxin content of *O*. cf. *ovata* cells under N- and P-limited conditions. Further research is needed to clarify how allelochemicals from macrophytes could influence dinoflagellate toxin production since an increase in the toxin contents could have important implications when using macrophytes as bloom mitigation agents.

### Dinoflagellate behavior and natural association with macrophytes

In our study, the magnoliophytes *Z*. *noltei* and *C*. *nodosa* induced a lower inhibitory effect compared to *U*. *rigida*. Previous *in situ* studies have reported that *Cymodocea* spp. and *Zostera* spp. represent typical host species and are usually colonized by epiphytes. Turki and El Abed [85], Turki [86], and Aligizaki et al. [87] have indeed reported a high abundance of *P*. *lima* cells on *C*. *nodosa* leaves. Foden et al. [88] have identically observed high densities of *P*. *lima* associated with *Zostera* beds. However, our results showed that among all the benthic dinoflagellates, *P*. *lima* was the most sensitive to the bioactivity of the tested magnoliophytes, with systematic significant responses whatever the macrophyte considered, whereas *O*. cf. *ovata* and *C*. *monotis* appeared sensitive only to the presence of *U*. *rigida* thalli. In addition to different physiological adaptations acquired along co-evolutionary processes, it can be hypothesized that the highest vulnerability of *P*. *lima* might be due to its distinct behavior in culture. *P*. *lima* cells were motionless and attached to the entire surface of the leaves, thus enhancing the cellular exposure to the allelochemicals. In contrast, *O*. cf. *ovata* and *C*. *monotis* were suspended in the water column and attached only to the edges of the magnoliophyte leaves. Direct contact between *P*. *lima* cells and macrophyte leaves may therefore promote the inhibitory effect. Our results suggest that dinoflagellate adhesion patterns should be also taken into account in order to better explain the observed allelopathic effects. Concerning *Ulva* thalli, field surveys showed contradictory observations. Low epiphytic dinoflagellate abundances have been reported [89,90], while moderate to high cell densities were also observed on the macroalgae depending on the species and the marine habitats studied [91–93]. Otherwise, it has been suggested that macrophyte morphotypes can drive host preference trends. Parsons and Preskitt [91] have thus found that *P*. *lima* and *C*. *monotis* preferred microfilamentous macroalgae, while *O*. *ovata* cells were more abundant on microblades thalli.

Nevertheless, it has been suggested [26,91] that epiphytic regulation and host preferences seem to depend mostly on the specific requirements of dinoflagellates and on the inhibitory efficiency of the allelochemicals released by the macrophytes.

### Chemicals potentially responsible for the observed allelopathic effects

Table 2 summarizes the known chemicals identified, produced, and released by *U*. *rigida*, *Z*. *noltei*, and *C*. *nodosa* species and their reported potential biological activity (See also S3 Appendix). From the data gathered in Tables 1 and 2 and in the S3 Appendix, we can hypothesize that the inhibitory effect caused by *Z*. *noltei* and *C*. *nodosa* on dinoflagellate species was mainly related to the production of polyphenols, whereas for *U*. *rigida*, polyunsaturated fatty acids (PUFAs) seem to be the most incriminated inhibitory compounds. However, the identification of these potential allelochemicals in our own macrophyte species and the evaluation of their inhibitory activity using biological tests are required.

**Table 2:** Phytochemicals associated with *Ulva rigida*, *Zostera noltei* and *Cymodocea nodosa* species with their reported biological activity.

Macrophyte species and origin	Detected and identified compounds	Reported biological activities	Reference
***Ulva rigida***			
*Ulva rigida* (Ras-Djebel, Tunisia)	**Polyphenols**: Phloroglucinol / Feruloyl-hexose / Fucodiphloroethol / Vanillic acid / Fucophloroethols derivatives / Quinin acid / Dieckol / Fucophloroethol / Syringic acid / Phloroeckol / Dihydroxybenzoic acid / Phenylethanol / Dioxinodehydroeckol / Eckol / Diphloroethohydroxycarmalol.	Radical-scavenging activity.	[105]
		Not toxic to HeLa cells culture.	
*Ulva rigida* (Ria Formosa, Portugal)	**Fatty acids**: Linoleic / α-linolenic / Stearidonic / γ-linolenic / Arachidonic / Eicosapentaenoic / Oleic / Palmitoleic.	Not Tested	[117]
	**Polyunsaturated aldehydes** (detected upon tissue damage): 2,4-Heptadienal / 2,4-decadienal / 2,4,7-decatrienal.		
*Ulva rigida* (Sidi Mansour, Sfax, Tunisia)	**Fatty acids:** Palmitic / Oleic / Linolenic / Eicosenoic / Linoleic / Palmitoleic / Stearic / Myristic /Arachidic.	Antibacterial, antimicrobial and antioxidant activities. Acetylcholinesterase inhibitory capacity.	[104]
*Ulva rigida* (Black Sea)	**Sterols:** Fucosterol (= main sterol component)	Not Tested	[118]
***Zostera noltei***			
*Zostera noltei* (Algarve, Southern Portugal)	**Phenolic acid:** Rosmarinic acid.	Radical scavenging activity. Capacity to chelate copper and iron ions. Toxicity against HepG2, S17 and neuroblastoma cell lines.	[119]
	**Fatty acids:** Palmitic / Linoleic / α-linolenic / Myristic / Margaric / Stearic / Arachidic / Behenic / Lignoceric / Palmitoleic / Oleic / Hexadecatrienoic / Arachidonic / Eicosapentaenoic / Docosahexaenoic.		
*Zostera noltii* (Thau lagoon and Arcachon bay, France)	**Phenolics:** Zosteric acid / Rosmarinic acid / Flavonoids.	Algicidal activity against the neuro-toxic dinoflagellate *Alexandrium catenella*.	[29]
*Zostera noltii* (Bays of Arcachon, France; Cadiz, Spain)	**Phenolics:** Zosteric acid / Caffeic acid **/** Luteolin 7-sulfate / Apigenin 7-glucoside / Apigenin 7- sulfate / Diosmetin 7-sulfate / Luteolin / Apigenin / Diosmetin.	Not Tested	[101]
*Zostera noltii* (Bays of Cadiz, Sa Nitja and Alfacs, Spain; Arcachon lagoon, France)	**Phenolics**: Rosmarinic acid / Zosteric acid / Caffeic acid.	Not Tested	[100]
*Zostera noltii* (Arcachon lagoon, France)	**Phenolics**: Rosmarinic acid / traces of Caffeic acid.	Not Tested	[99]
*Zostera noltii* (Arcachon lagoon, France)	**Phenolic acid:** Zosteric acid.	Not Tested	[98]
*Zostera noltii* (Spain)	**Phenolics:** *p*-Coumaric / *p*-Hydroxybenzoic acids.	Not Tested	[120]
*Zostera nana* (Bucknall; Isle of Wight, U.K)	**Two Flavone sulfates:** Luteolin 7-sulphates / Diosmetin.	Not Tested	[121]
***Cymodocea nodosa***			
*Cymodocea nodosa* (Chebba coast, Tunisia)	Sulfated polysaccharide	Anti-hypertensive properties.	[122]
*Cymodocea nodosa* (Gran Canaria, Canary Islands; Cadiz and Alfacs bays, Spain; Zeytineli, Turkey; Sahline Sebkha beach-Monastir, Tunisia)	**Phenolic acids:** Chicoric acid / Caftaric acid.	Not Tested	[50]
*Cymodocea nodosa* (Porto Germeno, Greece)	Deoxycymodienol / Isocymodiene / Meroterpenoid (nodosol) /Brominated briarane diterpene / Cymodienol	Antibacterial activity.	[49]
*Cymodocea nodosa* (Ag. Cosmas Gulf, Greece)	**Four 3-keto steroids:** (20*R*)-22*E*-24-ethylcholesta-4,22-dien-3-one / (20*R*)-24-ethylcholest-4-en-3-one / (20*R*)-22*E*-6β-hydroxy-24-ethylcholesta-4,22-dien-3-one **/** 6β-hydroxy-(20*R*)-24-ethylcholest-4-en-3-one.	No data	[102]
*Cymodocea nodosa* (Ag. Cosmas Gulf, Greece)	**Diarylheptanoids:** Cymodienol **/** Cymodiene.	Cytotoxic activity against two lung cancer cell lines (NSCL-N6 and A549).	[48]
*Cymodocea nodosa* (Bay of Naples, Italy)	**Sterols**: Most abundant compounds: Sitosterol / Cholesterol / Stigmasterol.	Not Tested	[123]
*Cymodocea nodosa* (Bat-Yam)	Sulfated phenolic acids	Not Tested	[124]
*Cymodocea nodosa* (Ganzirri, Sicily; Marsaxlokk, Malta)	1-*chiro*-inositol / *myo*-inositol / *muco*-inositol.	Not Tested	[125]

(Macrophyte species are named as cited in the references).

Phenolics play a key role in the defense strategy of plants against pathogens and herbivores [94]. They are also known to induce inhibitory effects on phytoplankton growth [20]. Several biological activities, including antioxidant and antimicrobial properties, have been attributed to polyphenols [95–97]. Laabir et al. [29] have reported that *Z*. *noltei* and *Z*. *marina* crude extracts, which inhibited the growth of *A*. *catenella*, contained significant amounts of phenolics (zosteric acid, rosmarinic acid, and flavonoids). Achamlale et al. [98,99] and Grignon-Dubois et al. [100] have found substantial concentrations of phenolic acids (rosmarinic, zosteric, and caffeic acids) in *Z*. *noltei* detrital leaves and in crude extracts. *Z*. *noltei* is also characterized by the presence of flavonoids [101]. Like other polyphenols, these compounds have the capacity to act as antioxidants and can also affect growth and important metabolic functions of harmful microalgal species, including cyanobacteria and dinoflagellates [74].

Less is known about allelochemicals produced by *C*. *nodosa* and evaluation of their bioactivity needs more clarification. Grignon-Dubois and Rezzonico [50] have screened detrital and fresh specimens and identified chicoric acid as the major polyphenol of *C*. *nodosa*. Kontiza et al. [48] and Kontiza et al. [102] have isolated two diarylheptanoids (cymodienol and cymodien) and four 3-keto steroids from *C*. *nodosa*, respectively. They reported moderate to strong cytotoxic activity of these compounds. Four other metabolites (deoxycymodienol, isocymodiene, nodosol, and briarane diterpene) have been isolated from the organic extract of this seagrass and exhibited weak to strong antibacterial activity [49]. Among the extracted compounds, cymodienol and nodosol were the most active substances.

For *Ulva* species, there is an increasing evidence that these macroalgae have a strong inhibitory allelopathic activity [70,103]. It has been reported that PUFAs produced by *Ulva* spp. have potent algicidal activity and can act as allelochemicals [52–53]. These compounds were highly active against several red tide phytoplankton species even at low concentrations [52–53]. Alamsjah et al. [52] have reported that PUFAs were released into the seawater and gradually decomposed with time. Antibacterial and antimicrobial activities were reported for *U*. *rigida*, and the fatty acid composition of this macroalgae was investigated [104]. Otherwise, *U*. *rigida* has shown biological activities that are related not only to fatty acids but also to the presence of polyphenols [105]. Reports dealing with the phytochemistry of *U*. *rigida* and the identification of its polyphenols remain scarce. Mezghani et al. [105] have reported that *U*. *rigida* extracts contained various polyphenols and they also noted the presence of phlorotannins such as phloroglucinol, a compound usually reported to occur in brown marine algae [106]. All of these molecules are well known for their potent antioxidant and radical scavenging activities, which confirms the inhibitory effects observed in our study.

## Conclusion

Our results highlight the potential of the macrophytes *Z*. *noltei*, *C*. *nodosa*, and *U*. *rigida* to reduce the proliferation of the HAB-forming benthic marine dinoflagellates *O*. cf. *ovata*, *P*. *lima*, and *C*. *monotis* but with contrasted efficiencies. We demonstrated that the planktonic *A*. *pacificum* can be strongly affected by the presence of *C*. *nodosa* and *U*. *rigida* fresh leaves/thalli. Our findings suggest that benthic dinoflagellates seem more resistant than planktonic species to potential allelochemicals released by the macrophytes. The variable sensitivity of target phytoplankton species to the same macrophyte provides some insights for a better understanding of the complex species-specific allelopathic interactions occuring in marine ecosystems. In freshwater ecosystems, an important feature associated with the allelopathical relationship between macrophytes and microalgae is related to their species-specific nature [107,108]. Some macrophytes are more potent than others and may exert stronger effects on phytoplankton [109,110], whereas differential sensitivities of phytoplankton taxa have been described [107,111]. Allelopathy might play a crucial role in determining species compositions and dominance patterns by regulating the diversity and structure of phytoplankton communities. Depending on the macrophyte species, inhibitory effects were observed on growth and photosynthesis. Cell toxin production also seemed to respond to the stress induced by the presence of the macrophytes.

Future investigations have to isolate and identify the allelopathic substances that are effectively exudated in the seawater by the macrophytes investigated in our study. Their modes of action and the physiological processes that are impacted, have also to be thoroughly explored for a potential use of macrophytes in bloom control and mitigation. A better understanding of the co-evolutionary relationships between epibenthic dinoflagellates and macrophytes would be of great interest, as these processes could explain the resistance or tolerance of the targeted species to allelochemicals released by the macrophytes with which they have co-evolved.

## Supporting information

S1 Appendix NO_3_^-^ and PO_4_^3-^ concentrations (μmol.L^-1^) in all experiments according to the macrophyte species. (DOCX)

S2 Appendix Dinoflagellate colonization patterns. (PDF)

S3 Appendix Phytochemicals associated with *Ulva*, *Zostera* and *Cymodocea* species with their reported biological activity. (DOCX)
