# Supplementary material for: New insights on the species-specific allelopathic interactions between macrophytes and marine HAB dinoflagellates
Source: PLoS One. 2017 Nov 17;12(11):e0187963. doi: 10.1371/journal.pone.0187963 (PMC5693406; doi:10.1371/journal.pone.0187963)
Supplement: S1 Appendix — (DOCX) [file pone.0187963.s001.docx]

**S1 Appendix. NO_3_^-^ and PO_4_^3-^ concentrations (µmol.L^-1^) in all experiments according to the macrophyte species**.

|  | | ***Zostera noltei*** | | | | | | | | | |
| --- | --- | --- | --- | --- | --- | --- | --- | --- | --- | --- | --- |
|  |  | Control | | 0.1 g | | 0.3 g | | 0.75 g | | 1.5 g | |
|  |  | **Day 0** | **Day 10** | **Day 0** | **Day 10** | **Day 0** | **Day 10** | **Day 0** | **Day 10** | **Day 0** | **Day 10** |
| **NO_3_^-^** | Mean  SD | 478.96  6.42 | 372.61  13.73 | 473.84  5.53 | 356.03  10.58 | 468.6  4.49 | 349.63  14.58 | 460.19  8.07 | 284.21  21.75 | 444 .53  9.77 | 237.16  17.88 |
| **PO_4_^3-^** | Mean  SD | 17.43  0.73 | 9.88  1.06 | 16.99  0.53 | 9.76  0.85 | 16.84  0.44 | 9.42  0.72 | 16.83  0.48 | 10.05  0.96 | 16.92  0.53 | 9.09  0.64 |
|  | | ***Cymodocea nodosa*** | | | | | | | | | |
|  |  | Control | | 0.1 g | | 0.3 g | | 0.75 g | | 1.5 g | |
|  |  | **Day 0** | **Day 10** | **Day 0** | **Day 10** | **Day 0** | **Day 10** | **Day 0** | **Day 10** | **Day 0** | **Day 10** |
| **NO_3_^-^** | Mean  SD | 480.91  10.00 | 376.60  13.53 | 473.58  6.01 | 345.67  10.45 | 460.37  5.67 | 321.87  10.69 | 451.63  10.03 | 285.13  21.93 | 439.92  9.55 | 238.79  18.62 |
| **PO_4_^3-^** | Mean  SD | 17.32  0.82 | 9.88  0.78 | 17.62  0.92 | 9.58  0.68 | 17.97  0.61 | 9.79  0.99 | 17.42  0.81 | 9.27  0.63 | 17.19  0.72 | 9.38  0.69 |
|  | | ***Ulva rigida*** | | | | | | | | | |
|  |  | Control | | 0.08 g | | 0.16 g | | 0.5 g | | 1.0 g | |
|  |  | **Day 0** | **Day 10** | **Day 0** | **Day 10** | **Day 0** | **Day 10** | **Day 0** | **Day 10** | **Day 0** | **Day 10** |
| **NO_3_^-^** | Mean  SD | 484.40  7.18 | 370.84  10.63 | 476.69  10.73 | 324.94  21.78 | 463.74  11.07 | 297.66  20.92 | 447.02  9.82 | 255.34  18.31 | 418.49  7.25 | 192.42  10.89 |
| **PO_4_^3-^** | Mean  SD | 17.32  0.62 | 9.33  0.86 | 17.47  0.60 | 9.05  0.66 | 17.21  0.67 | 9.57  0.85 | 17.01  0.64 | 9.20  0.90 | 16.95  0.50 | 9.71  0.85 |

Means and Standard Deviations for each treatment at the beginning (Day 0) and the end of the experiments (Day 10); N=9 for *Zostera noltei* and N=12 for *Cymodocea nodosa* and *Ulva rigida*.
