# Supplementary material for: New insights on the species-specific allelopathic interactions between macrophytes and marine HAB dinoflagellates
Source: PLoS One. 2017 Nov 17;12(11):e0187963. doi: 10.1371/journal.pone.0187963 (PMC5693406; doi:10.1371/journal.pone.0187963)

O.* cf. *ovata - Z. noltei

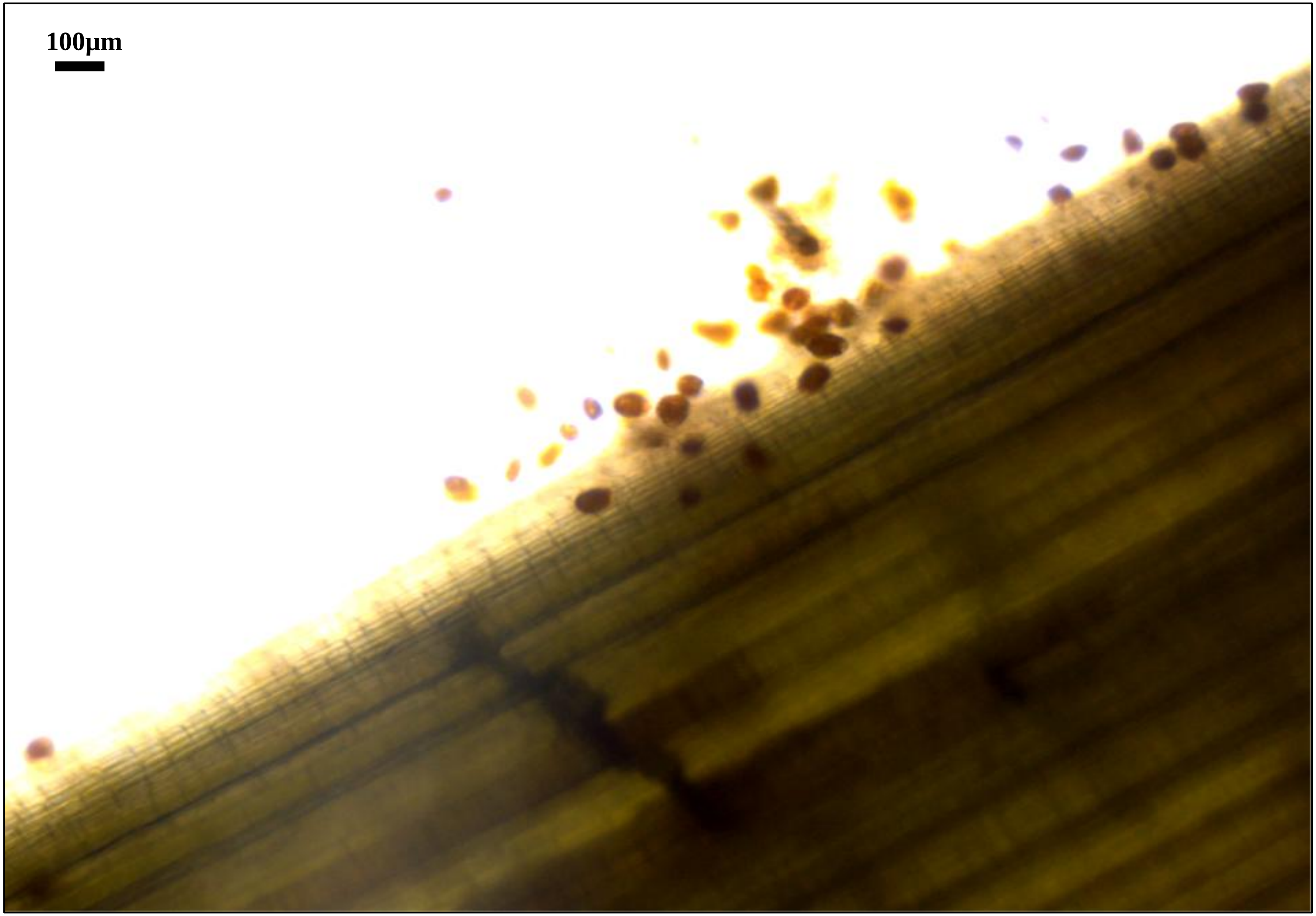

O.* cf. *ovata - C. nodosa

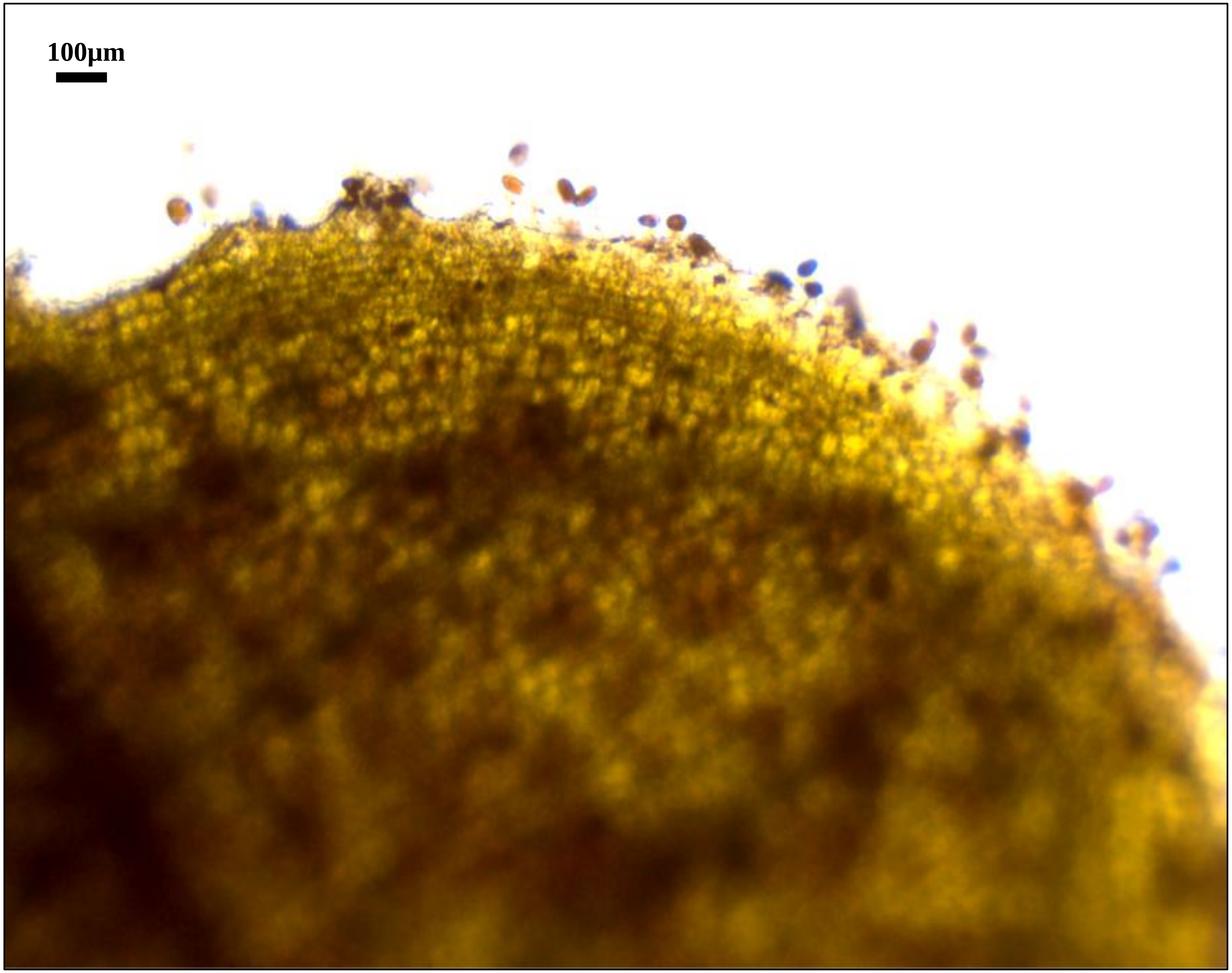

O.* cf. *ovata - U. rigida

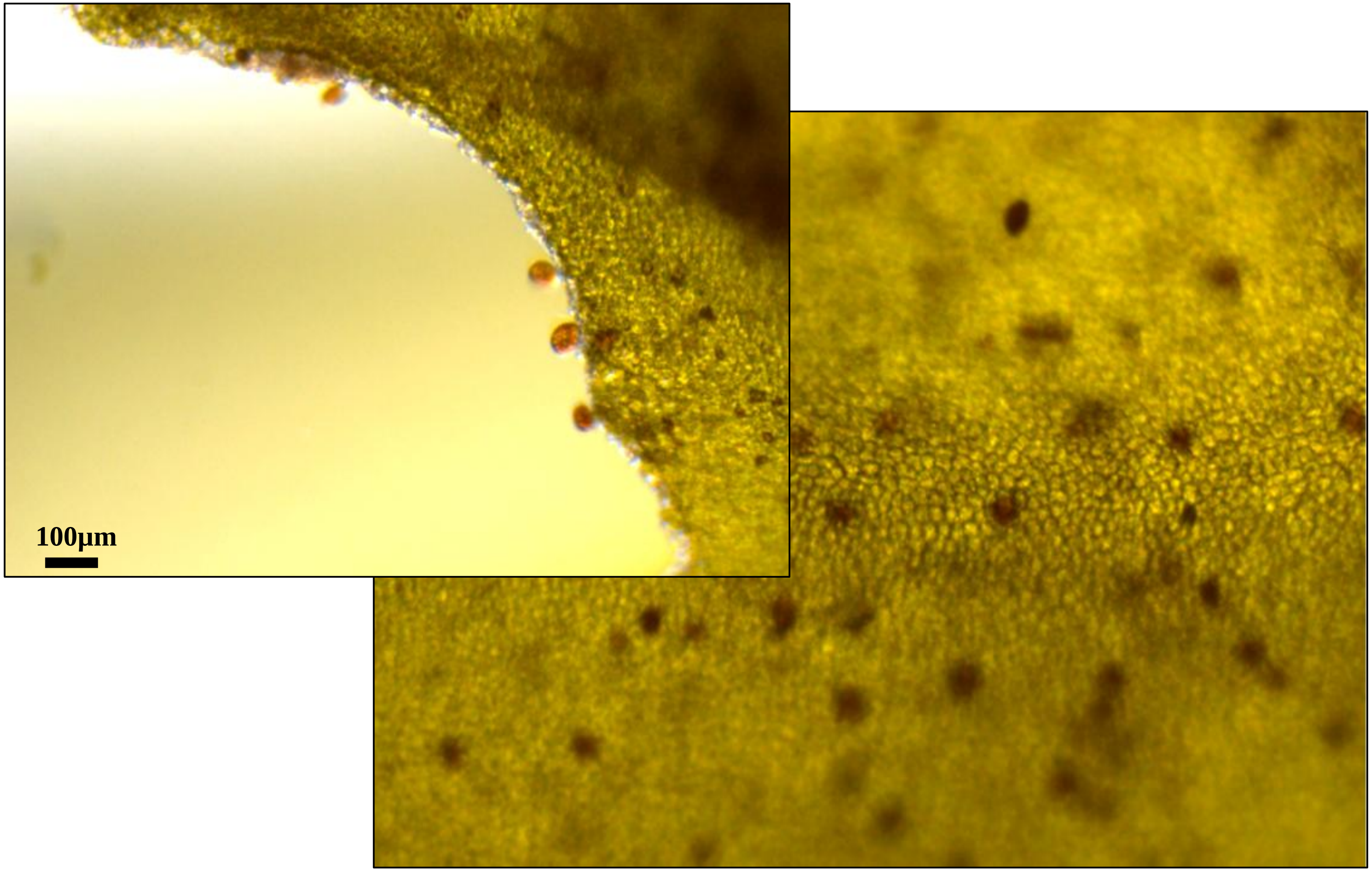

P. lima - Z. noltei

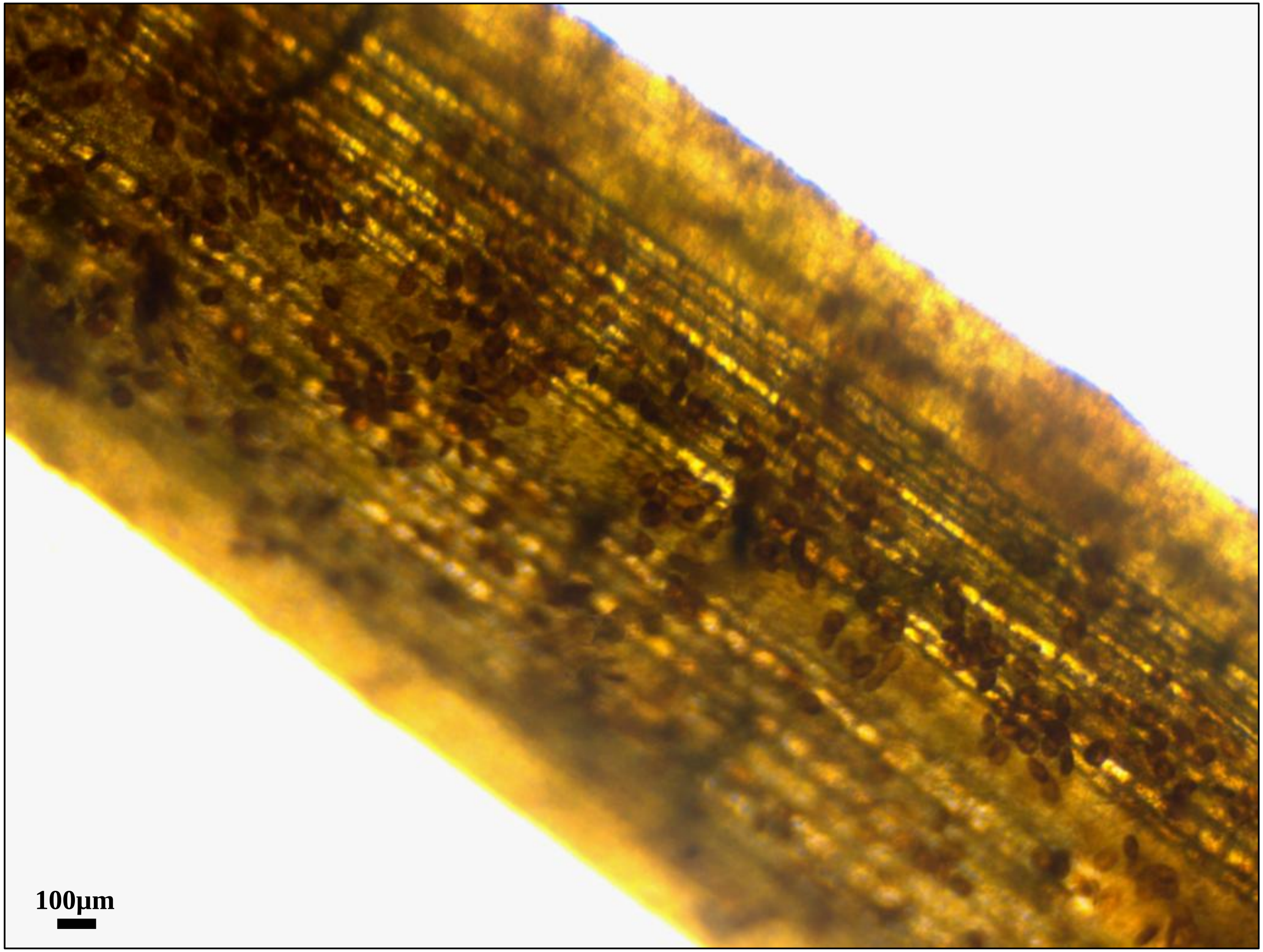

P. lima - C. nodosa

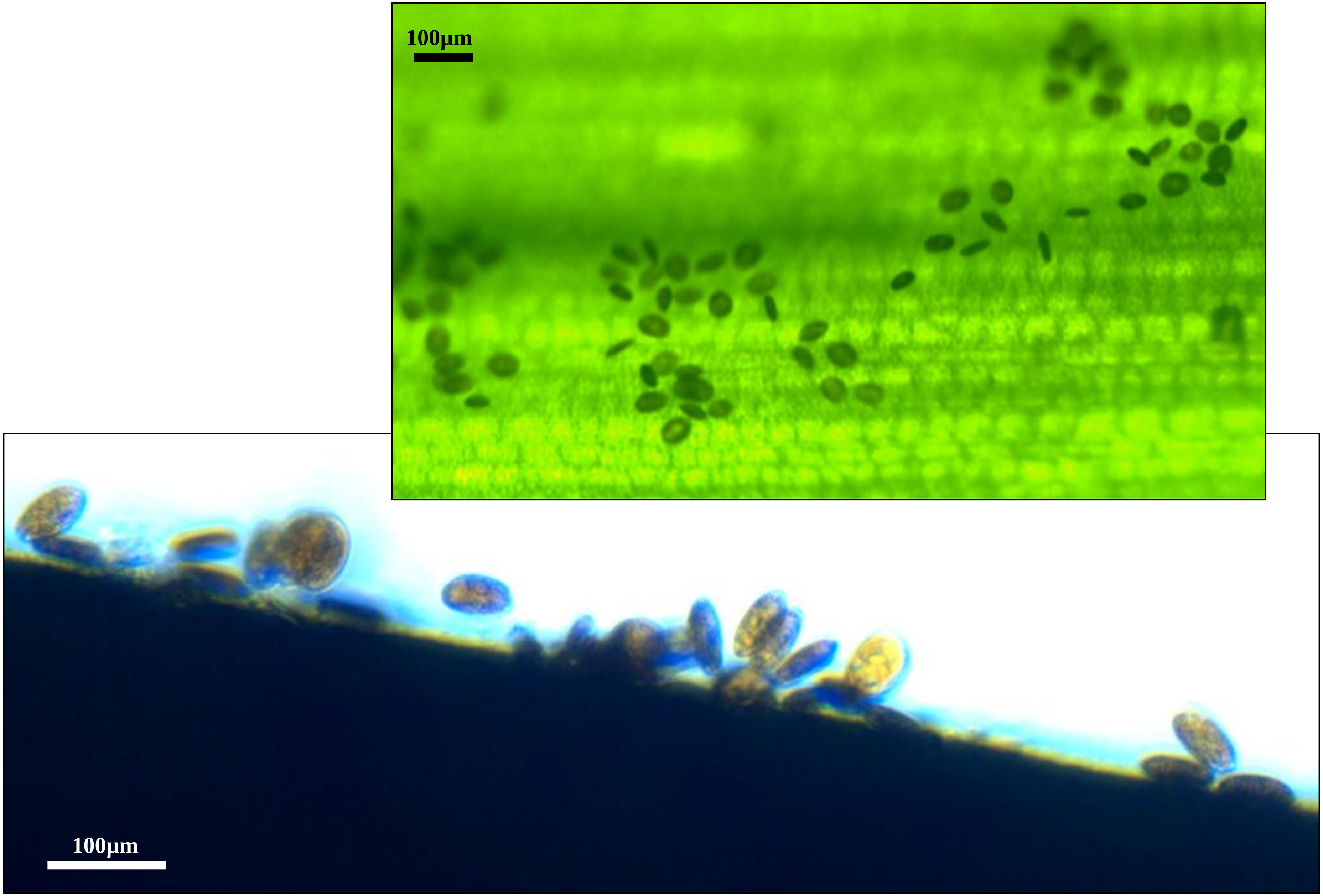

P. lima - U. rigida

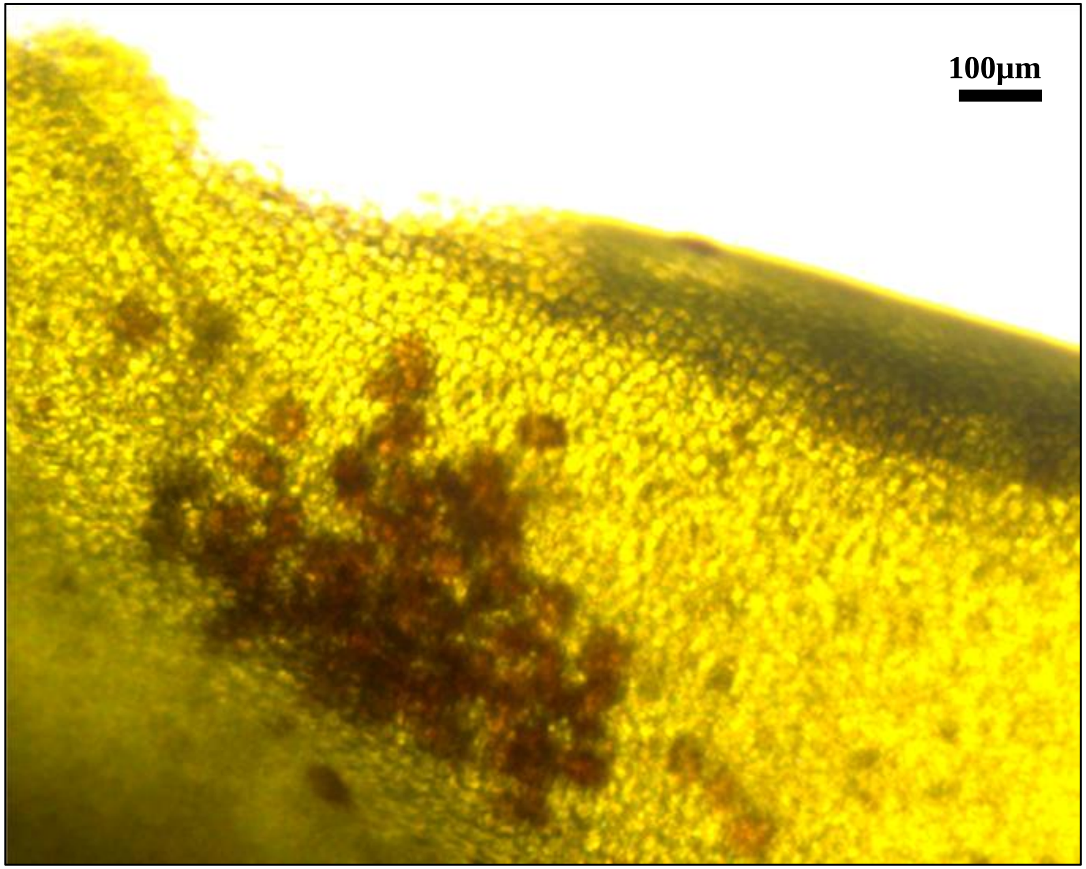

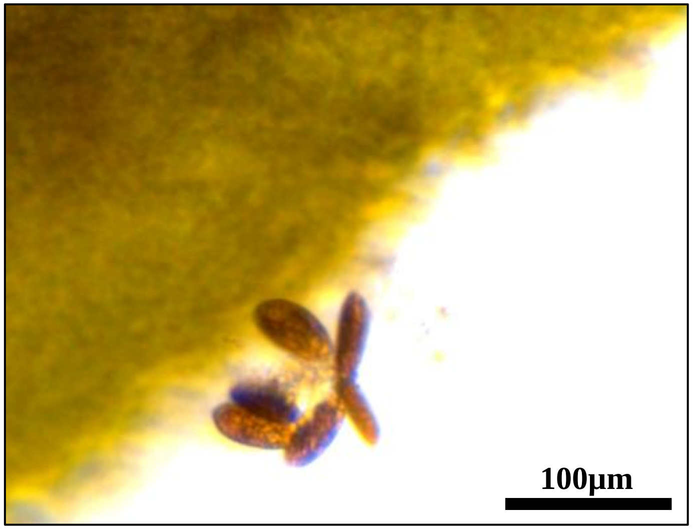

C. monotis - Z. noltei

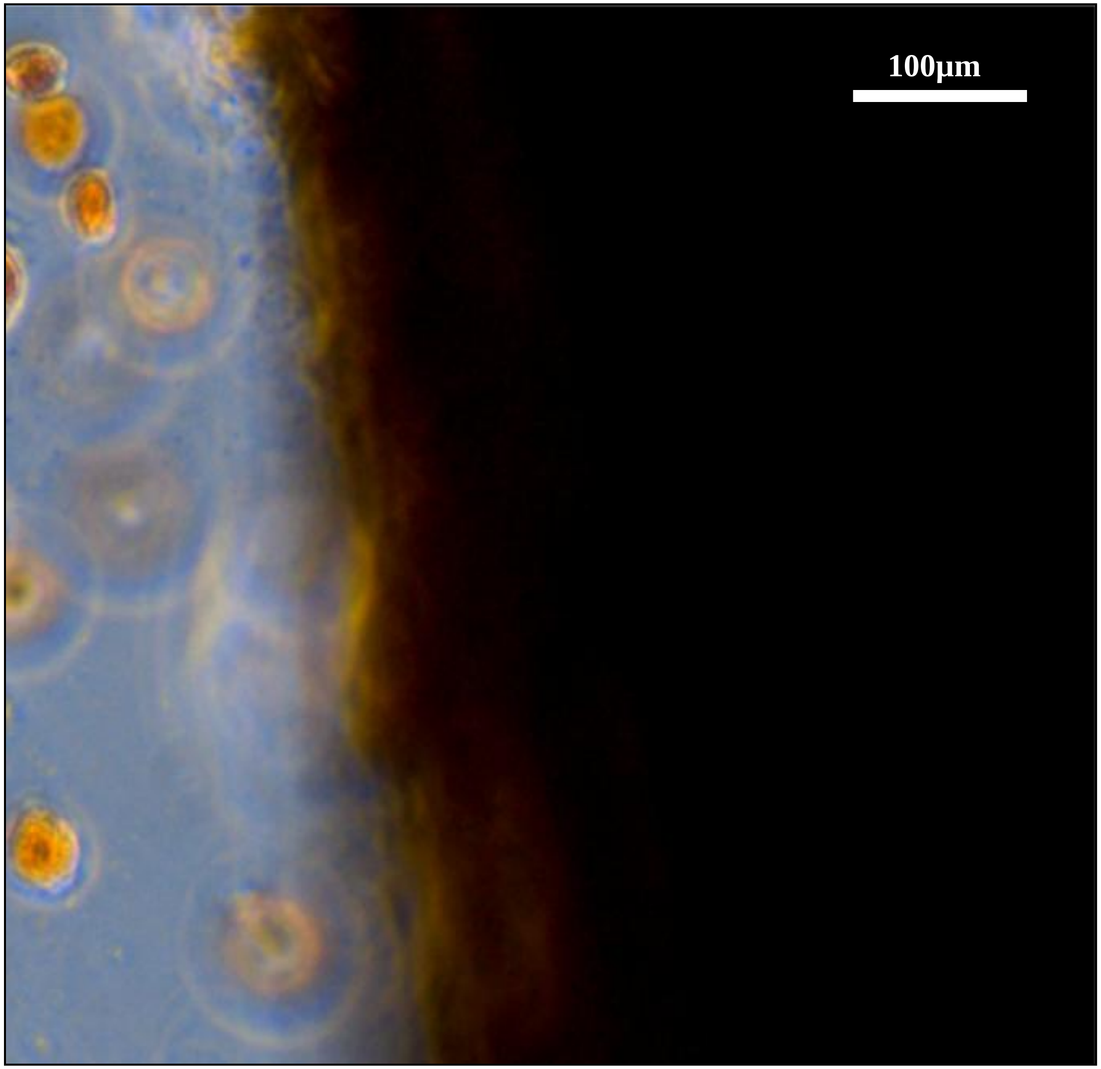

C. monotis - C. nodosa

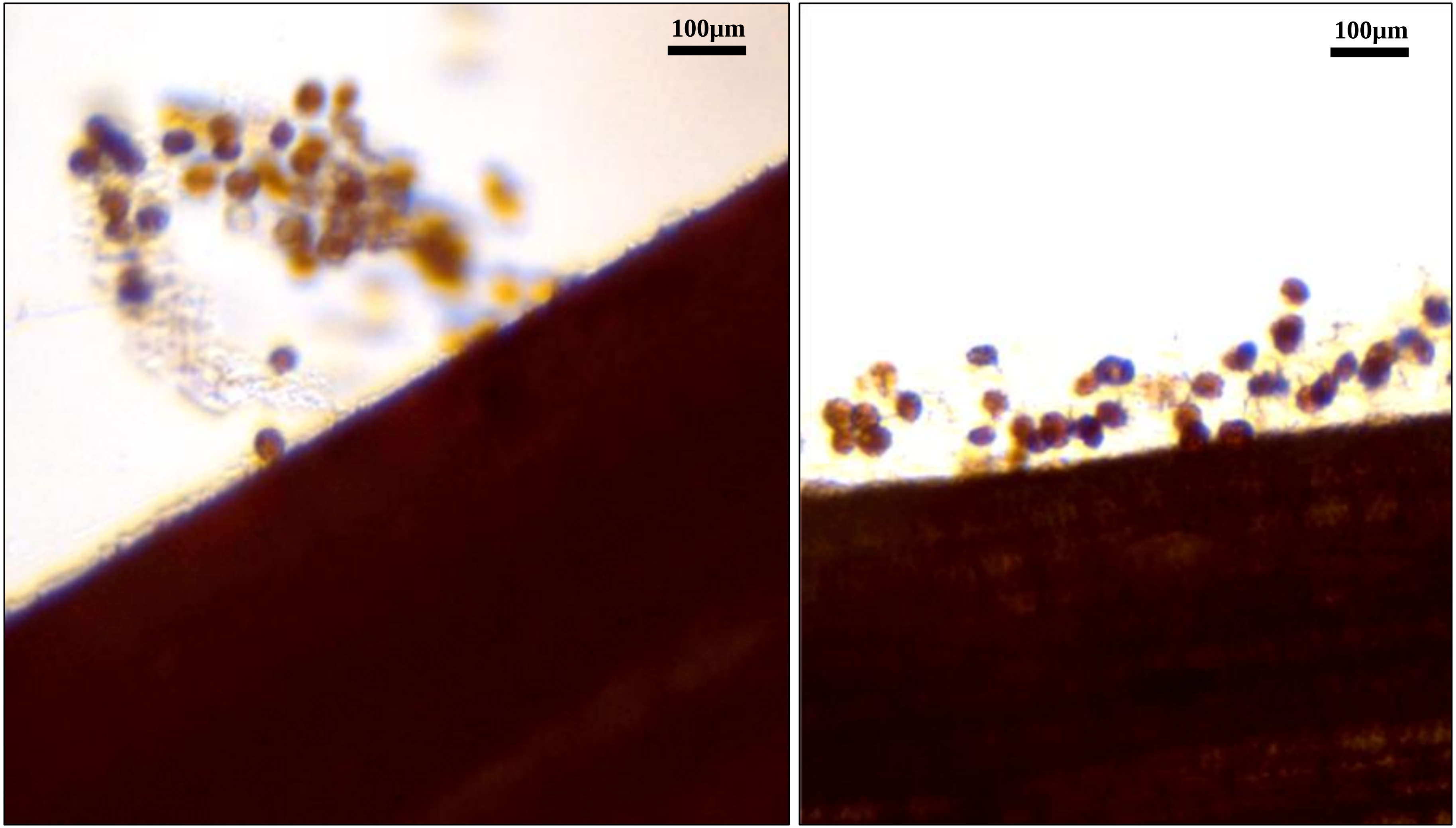

C. monotis - U. rigida

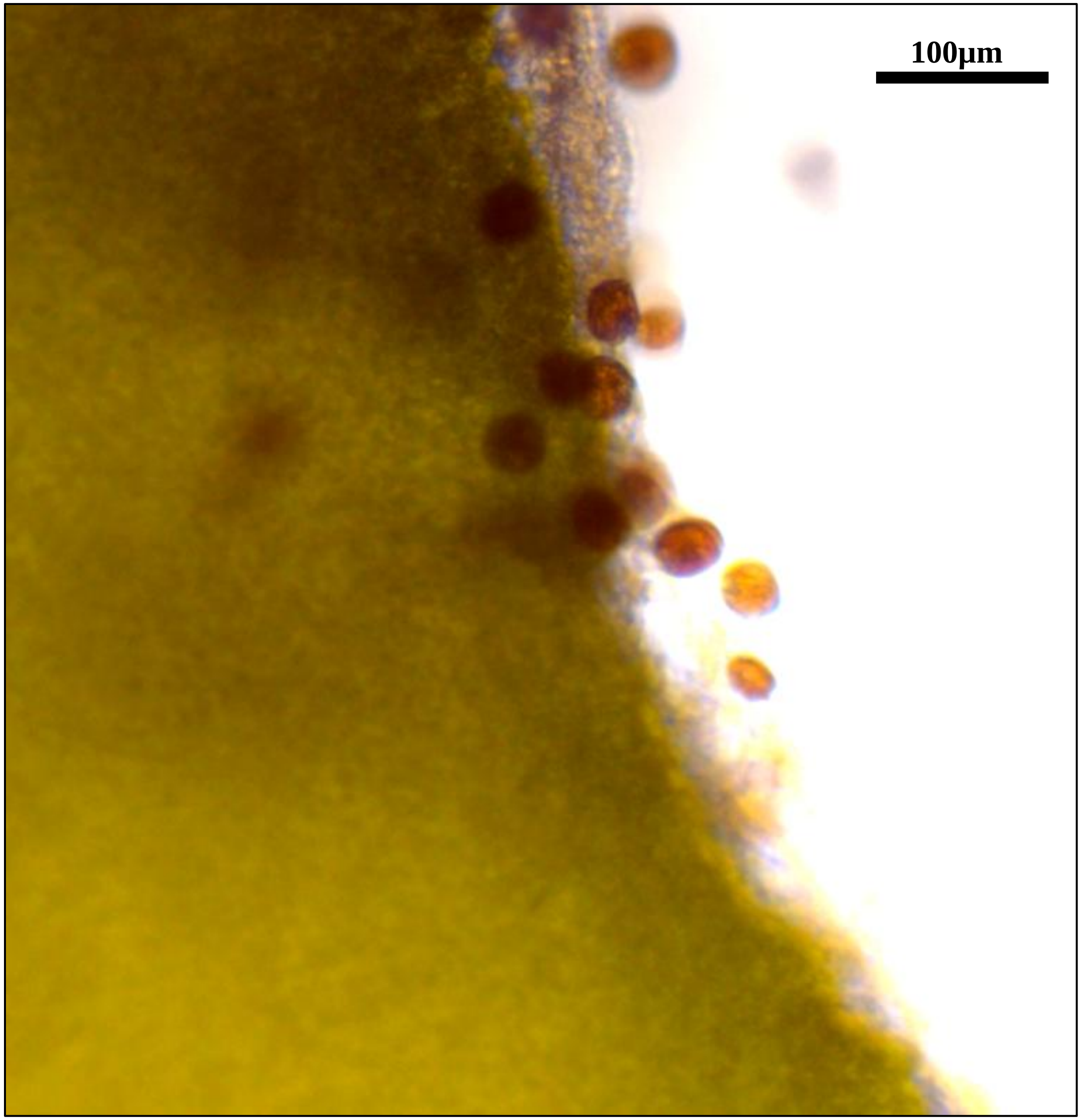

Supplement: S2 Appendix — (PDF) [file pone.0187963.s002.pdf]
