# Supplementary material for: New insights on the species-specific allelopathic interactions between macrophytes and marine HAB dinoflagellates
Source: PLoS One. 2017 Nov 17;12(11):e0187963. doi: 10.1371/journal.pone.0187963 (PMC5693406; doi:10.1371/journal.pone.0187963)
Supplement: S3 Appendix — (DOCX) [file pone.0187963.s003.docx]

**S3 Appendix:** Phytochemicals associated with *Ulva, Zostera* and *Cymodocea* species with their reported biological activity.

| **Macrophyte species and origin** | **Detected and identified compounds** | **Reported biological activities** | **Reference** |
| --- | --- | --- | --- |
| ***Ulva* spp.** | | | |
| *Ulva rigida*  (Ras-Djebel, Tunisia) | **Polyphenols**: Phloroglucinol / Feruloyl-hexose / Fucodiphloroethol / Vanillic acid / Fucophloroethols derivatives / Quinin acid / Dieckol / Fucophloroethol / Syringic acid / Phloroeckol / Dihydroxybenzoic acid / Phenylethanol / Dioxinodehydroeckol / Eckol / Diphloroethohydroxycarmalol. | Radical-scavenging activity.  Not toxic to HeLa cells culture. | Mezghani et al. 2016 |
| *Ulva intestinalis*  (Fenghua coast, China) | **Three algicidal compounds:** 15-ethoxy-(6z,9z,12z)-hexadecatrienoic acid / (6E,9E,12E)-(2-acetoxy-*β*-*D*-glucose)-octadecatrienoic acid ester / Hexadecanoic acid. | Algicidal activity against red tide microalgae | Sun et al. 2016 |
| *Ulva rigida*  (Ria Formosa, Portugal) | **Fatty acids**: Linoleic / α-linolenic / Stearidonic / γ-linolenic / Arachidonic / Eicosapentaenoic / Oleic / Palmitoleic.  **Polyunsaturated aldehydes** (detected upon tissue damage): 2,4-Heptadienal / 2,4-decadienal / 2,4,7-decatrienal. | Not Tested | Alsufyani et al. 2014 |
| *Ulva rigida*  (Sidi Mansour, Sfax, Tunisia) | **Fatty acids:** Palmitic / Oleic / Linolenic / Eicosenoic / Linoleic / Palmitoleic / Stearic / Myristic /Arachidic. | Antibacterial, antimicrobial and antioxidant activities. Acetylcholinesterase inhibitory capacity. | Trigui et al. 2013 |
| *Ulva fasciata*  (South-West coast of India) | **Seven labdane diterpenoids**: Labda-14-ene-8-ol / labda-14-ene-3α,8α-diol / Labda-14-ene-8α,9α-diol  / Labda-14-ene-8α-hydroxy-3-one / ent-Labda-13(16),14-diene-3-one / ent-Labda-13(16),14-diene / ent-Labda-13(16),14-diene-3α-ol. | Antibacterial activity. | Chakraborty et al. 2010 |
| *Ulva fasciata*  (South-Western India) | **Five major sesquiterpenoids:** 2,5,5-Trimethyl-4-(4’-methyl-3’-pentenyl)-2-cyclohexen-1-ol / 4-Isopentyl-3,4,5,5-tetramethyl-2-cyclohexen-1-ol / 4-Isopentyl-3,4,5,5-tetramethyl-2-cyclohexen-1-ol / 6-Isopentyl-1,5,5,6-tetramethyl-1-cyclohexene / 3,4,5,5-Tetramethyl-4-(3’-oxopentyl)-2-cyclohexen-1-one. | Radical-scavenging activity. | Chakraborty and Paulraj 2010 |
| *Ulva fasciata*  (Abu-Qir, Egypt) | **Three new fatty acids**: (*E*)-11-oxo-octadeca-12-enoic acid / (*E*)-11-hydroxy-octadeca-12-enoic acid / 6-hydroxy-oct-7-enoic acid.  **Unpolar fractions**: Dimethylsulfoxide / Dimethylsulfone / 4-Oxo-pentanoic acid / Dodecane / Tridecane / 1,1*’-*Bicyclohexyl / Phenylacetamide / 6,10,14-Trimethyl-pentadecan-2-one / 8-Heptadecene / Hexadecanoic acid. | Antimicrobial activity. Weak cytotoxicity against brine shrimps. Potent antitumor activity against breast carcinoma tumor cell line MCF7. | Abou-Elwafa et al. 2009 |
| *Ulva fasciata, Ulva pertusa,*  *Ulva arasakii, Ulva conglobota* (Nagasaki, Japan) | **Three algicidal compounds = Polyunsaturated fatty acids (PUFAs):** Hexadeca-4,7,10,13-tetraenoic acid **/** Octadeca-6,9,12,15-tetraenoic acid **/** α-linolenic acid.  **Other Fatty acids:** Decanoic / Myristic / Myristoleic / Palmitic / Stearic / Elaidic / Oleic / Linolic / Eicosenoic / Behenic / Erucic. | Algicidal activity against phytoplankton species. | Alamsjah et al. 2008 |
| *Ulva fasciata*  (**) | **Fatty acids:** α-linolenic acid and linoleic acid. | Algicidal activity against red tide microalgae. Toxic to the rotifer *Brachionus plicatilis*.  Low toxicity against fish (*Inimicus japonicus*), brine shrimp (*Artemia sp.*) and mammalian cell lines (U937, HeLa, Vero, and CHO cells). | Alamsjah et al. 2007 |
| *Ulva fasciata*  (Coast of Nagasaki Prefecture, Japan) | **Three algicidal compounds = Polyunsaturated fatty acids (PUFAs):** Hexadeca-4,7,10,13-tetraenoic acid **/** Octadeca-6,9,12,15-tetraenoic acid **/** α-linolenic acid. | Algicidal activity against red tide microalgae. | Alamsjah et al. 2005 |
| *Ulva lactuca*  (Abou-Kir, Egypt) | **Steroid:** 3-O-β-D glucopyranosyl-stigmasta-5,25-dien (3-O-β-D-glucopyranosyl clerosterol). | Antiinflammatory and antimicrobial activities. | Awad et al. 2000 |
| *Ulva curvata*  (Shem Creek, Charleston Harbor, USA) | **Enzyme:** Dimethylsulfoniopropionate (DMSP) lyase (responsible for producing dimethylsulfide in marine environments) | Not Tested | De Souza et al. 1996 |
| *Ulva rigida*  (Black Sea) | **Sterols:** Fucosterol (= main sterol component). | Not Tested | Popov et al. 1985 |
| ***Zostera* spp.** | | | |
| *Zostera noltei*  *Zostera marina*  (Algarve, Southern Portugal) | **Phenolic acid:** Rosmarinic acid.  **Fatty acids:** Palmitic / Linoleic / α-linolenic / Myristic / Margaric / Stearic / Arachidic / Behenic / Lignoceric / Palmitoleic / Oleic / Hexadecatrienoic / Arachidonic / Eicosapentaenoic / Docosahexaenoic acids.  Pentadecanoic acid detected only in *Z. marina*. | Radical scavenging activity.  *Z. marina* :Capacity to chelate copper  *Z. noltei*: Capacity to chelate copper and iron ions.  Toxicity against HepG2, S17 and neuroblastoma cell lines. | Custόdio et al. 2016 |
| *Zostera noltii , Zostera marina*  (Thau lagoon and Arcachon bay, France) | **Phenolics:** Zosteric acid / Rosmarinic acid / Flavonoids. | Algicidal activity against the neuro-toxic bloom-forming dinoflagellate *Alexandrium catenella*. | Laabir et al. 2013 |
| *Zostera marina*  (Qingdao, China) | **Phenolic acid:** Rosmarinic acid. | Nematicidal and antibacterial activities against PWN (Pine Wood Nematode) and its carrying bacteria. | Wang et al. 2012 |
| *Zostera noltii* (Bays of Arcachon, France; Cadiz, Spain) | **Phenolics:** Zosteric acid / Caffeic acid **/** Luteolin 7-sulfate / Apigenin 7-glucoside / Apigenin 7- sulfate / Diosmetin 7-sulfate / Luteolin / Apigenin / Diosmetin. | Not Tested | Grignon-Dubois and Rezzonico 2012 |
| *Zostera noltii* (Bays of Cadiz, Sa Nitja and Alfacs, Spain ; Arcachon lagoon, France) | **Phenolics**: Rosmarinic acid / Zosteric acid / Caffeic acid. | Not Tested | Grignon-Dubois et al. 2012 |
| *Zostera noltii*  *Zostera marina*  (Arcachon lagoon, France) | **Phenolics**: Rosmarinic acid / traces of Caffeic acid. | Not Tested | Achamlale et al. 2009a |
| *Zostera noltii*  *Zostera marina*  (Arcachon lagoon, France) | **Phenolic acid:** Zosteric acid. | Not Tested | Achamlale et al. 2009b |
| *Zostera marina*  (Roscoff, France) | **Phenolics**: Gallic acid/ Caffeic acid / *p*-coumaric acid/ Ferulic acid. | Caffeic acid: Potential growth-limiting properties against *Labyrinthula zosterae.* | Vergeer and Develi 1997 |
| *Zostera marina*  (Øresund, Denmark) | **Phenolics**: Rosmarinic acid / Caffeic acid. | Not Tested | Ravn et al. 1994 |
| *Zostera marina*  (Monterey Bay, California) | **Phenolic acid:** *p*-(sulphooxy) Cinnamic acid. | Antifouling activity. | Todd et al. 1993 |
| *Zostera marina*  (Great Bay, USA) | **Phenolics:** Caffeic acid derivative / Luteolin-7,3'-disulfate / Luteolin-7- sulfate / other flavones. | Not Tested. Potential growth-limiting properties against *Labyrinthula* "P." | Buchsbaum et al. 1990 |
| *Zostera marina*  (Puget Sound, Washington, USA) | **Phenolics**: Ferulic / Vanillic / *p*-hydroxybenzoic / Caffeic / Gallic / Protocatechuic / Gentisic acids. | Not Tested | Quackenbush et al. 1986 |
| *Zostera marina*  (Roberts Bank, Canada) | **Phenolics**: Caffeic / Protocatechuic / Gentisic (=not detected or isolated, pure phenolic acids tested) | Algicidal activity against the microalgae (*Platymonas sp*.). Antibacterial activity.  Amphipod grazing inhibition. | Harrison et al. 1982 |
| *Zostera noltii* (Spain) | **Phenolics:** *p*-Coumaric / *p*-Hydroxybenzoic. | Not Tested | Zapata and McMillan 1979 |
| *Zostera marina* (Washington) | **Phenolics:** Caffeic / Protocatechuic / *p*-Coumaric / *p*-Hydroxybenzoic / Ferulic / Vanillic / Gentisic / Gallic. | Not Tested |  |
| *Zostera marina* (Plymouth, U.K) | **Five Flavone sulfates:** 7-sulphates of Apigenin, Luteolin, Diosmetin and Chrysoeriol / 7.3’-disulphate of luteolin | Not Tested | Harborne and Williams 1976 |
| *Zostera nana* (Bucknall; Isle of Wight, U.K) | **Two Flavone sulfates:** Luteolin 7-sulphates / Diosmetin. | Not Tested |  |
| ***Cymodocea* spp.** | | | |
| *Cymodocea nodosa*  (Chebba coast, Tunisia) | Sulfated polysaccharide | Anti-hypertensive properties. | Ben Abdallah Kolsi et al. 2016 |
| *Cymodocea serrulata*  (Coast of Tuticorin, India) | Phenyl thioketone | Antibacterial activity. | Gnanambal et al. 2015 |
| *Cymodocea serrulata*  (Arockiapuram coast, India) | **Fatty acids and related esters**: 2-pentadecanone, 6,10,14-trimethyl / 1,2-benzenedicarboxylic acid butyl 1,2-methylpropyl ester / octadecanoic acid methyl ester / 1,2-benzenedicarboxylic acid diisooctyl ester / Oleic acid / Erucic acid. | Antibacterial and antimicroalgal activities. Antimacrofouling properties. | Iyapparaj et al. 2014 |
| *Cymodocea nodosa* (Gran Canaria, Canary Islands; Cadiz and Alfacs bays, Spain; Zeytineli, Turkey; Sahline Sebkha beach, Tunisia) | **Phenolic acids:** Chicoric acid / Caftaric acid. | Not Tested | Grignon-Dubois and Rezzonico 2013 |
| *Cymodocea rotundata*  *Cymodocea serrulata*  (Chinnapallam, India) | Coumarins / Flavonoids / Phenols / Proteins / Free AminoAcids / Quinones / Saponins / Sterols / Sugars / Terpenoids. | Antibacterial, cytotoxic and haemolytic activities. | Ragupathi Raja Kannan et al. 2013a |
| *Cymodocea rotundata*  *Cymodocea serrulata*  (Chinnapallam, India) | Phenol / Flavonoids / Tannin / Vitamin C / Vitamin E.  Characterized Phenolic compound = *p*-coumaric acid | Antioxidant activity. | Ragupathi Raja Kannan et al. 2013b |
| *Cymodocea rotundata*  (Coast of Tuticorin, India) | Tannins / Saponins / Resins / Proteins / Acidic Compounds / Reducing Sugar / Terpenoids / Cardiac Glycoside / Alkaloids. | Antibacterial activity. | Mani et al. 2012 |
| *Cymodocea serrulata*  (Coast of Thondi, India) | Alkaloids / Flavonoids / Phenols / Steroids / Tannins. | Antibacterial activity. | Ravikumar et al. 2011 |
| *Cymodocea nodosa*  (Porto Germeno, Greece) | Deoxycymodienol / Isocymodiene / Meroterpenoid (nodosol) /Brominated briarane diterpene / Cymodienol. | Antibacterial activity. | Kontiza et al. 2008 |
| *Cymodocea nodosa*  (Ag. Cosmas Gulf, Greece) | **Four 3-keto steroids:** (20*R*)-22*E*-24-ethylcholesta-4,22-dien-3-one / (20*R*)-24-ethylcholest-4-en-3-one / (20*R*)-22*E*-6β-hydroxy-24-ethylcholesta-4,22-dien-3-one **/** 6β-hydroxy-(20*R*)-24-ethylcholest-4-en-3-one. | No data | Kontiza et al. 2006 |
| *Cymodocea nodosa*  (Ag. Cosmas Gulf, Greece) | **Diarylheptanoids :** Cymodienol  **/** Cymodiene. | Cytotoxic activity against two lung cancer cell lines (NSCL-N6 and A549). | Kontiza et al. 2005 |
| *Cymodocea serrulata*  (Queensland, Australia) | **5 Sterols**: Most abundant compounds = 24-ethylcholesta-5,22E-dien-3β-ol (stigmasterol) / 24-ethylcholest-5-en-3β-ol (sitosterol).  **28** **Fatty acids:** Most abundant compounds =Linolenic acid / Palmitic acid / Linoleic acid. | Not Tested | Gillan et al. 1984 |
| *Cymodocea nodosa*  (Bay of Naples, Italy) | **Sterols**: Most abundant compounds: Sitosterol / Cholesterol / Stigmasterol. | Not Tested | Sica et al. 1984 |
| *Cymodocea nodosa,* *Cymodocea rotundata, Cymodocea serrulata*  (Different origins) | Sulfated phenolic acids | Not Tested | McMillan et al. 1980 |
| *Cymodocea rotundata* (Australia) *Cymodocea serrulata* (Kenya) | **Phenolic acids:** Caffeic / Protocatechuic / *p*-Coumaric / *p*-Hydroxybenzoic / Ferulic / Vanillic / Gentisic / Gallic. | Not Tested | Zapata and McMillan 1979 |
| *Cymodocea nodosa* (Ganzirri, Sicily; Marsaxlokk, Malta) | 1-*chiro*-inositol / *myo*-inositol / *muco*-inositol. | Not Tested | Drew 1978 |

(Macrophyte species are named as cited in the references)

**References:**

- Abou-Elwafa GSE, Shaaban M, Shaaban KA, El-Naggar MEE, Laatsch H. Three new unsaturated fatty acids from the marine green alga *Ulva fasciata* Delile. Z Naturforsch B J Chem Sci. 2009; 64: 1199-1207.
- Achamlale S, Rezzonico B, Grignon-Dubois M. Rosmarinic acid from beach waste: Isolation and HPLC quantification in *Zostera* detritus from Arcachon lagoon. Food Chem. 2009a; 113: 878-883.
- Achamlale S, Rezzonico B, Grignon-Dubois M. Evaluation of *Zostera* detritus as a potential new source of zosteric acid. J Appl Phycol. 2009b; 21: 347-352.
- Alamsjah MA, Hirao S, Ishibashi F, Oda T, Fujita Y. Algicidal activity of polyunsaturated fatty acids derived from *Ulva fasciata* and *U. pertusa* (Ulvaceae, Chlorophyta) on phytoplankton. J Appl Phycol. 2008; 20: 713-720.
- Alamsjah MA, Ishibe K, Kim D, Yamaguchi K, Ishibashi F, Fujita Y, et al. Selective Toxic Effects of Polyunsaturated Fatty Acids Derived from *Ulva fasciata* on Red Tide Phyotoplankter Species. Biosci Biotechnol Biochem. 2007; 71 (1): 265-268.
- Alamsjah MA, Hirao S, Ishibashi F, Fujita Y. Isolation and structure determination of algicidal compounds from *Ulva fasciata*. Biosci Biotechnol Biochem. 2005; 69: 2186-2192.
- Alsufyani T, Engelen AH, Diekmann OE, Kuegler S, Wichard T. Prevalence and mechanism of polyunsaturated aldehydes production in the green tide forming macroalgal genus *Ulva* (Ulvales, Chlorophyta). Chem Phys Lipids. 2014; 183: 100-109.
- Awad NE. Biologically active steroid from the green alga *Ulva lactuca*. Phytother Res. 2000; 14: 641-643.
- Ben Abdallah Kolsi R, Fakhfakh J, Krichen F, Jribi I, Chiarore A, Patti FP, et al. Structural characterization and functional properties of antihypertensive *Cymodocea nodosa* sulfated polysaccharide. Carbohydr Polym. 2016; 151: 511-522.
- Buchsbaum RN, Short FT, Cheney DP. Phenolic-nitrogen interactions in eelgrass, *Zostera marina* L.: possible implications for disease resistance. Aquat Bot. 1990; 37: 291-297.
- Chakraborty K, Lipton AP, Paul Raj R, Vijayan KK. Antibacterial labdane diterpenoids of *Ulva fasciata* Delile from southwestern coast of the Indian Peninsula. Food Chem. 2010; 119: 1399-1408.
- Chakraborty K, Paulraj R. Sesquiterpenoids with free-radical-scavenging properties from marine macroalga *Ulva fasciata* Delile. Food Chem. 2010; 122: 31-41.
- Custόdio L, Laukaityte S, Engelen AH, Rodrigues MJ, Pereira H, Vizetto-Duarte C, et al. A comparative evaluation of biological activities and bioactive compounds of the seagrasses *Zostera marina* and *Zostera noltei* from southern Portugal. Nat Prod Res. 2016; 30 (6): 724-728.
- De Souza MP, Chen YP, Yoch DC. Dimethylsulfoniopropionate lyase from the marine macroalga *Ulva curvata*: purification and characterization of the enzyme. Planta. 1996; 199: 433-438.
- Drew EA. Carbohydrate and Inositol metabolism in the seagrass, *Cymodocea nodosa*. New Phytol. 1978; 81: 249-264.
- Gillan FT, Hogg RW, Drew EA. The sterol and fatty acid compositions of seven tropical seagrasses from North Queensland, Australia. Phytochemistry. 1984; 23: 2817-2821.
- Gnanambal KME, Patterson J, Patterson EJK. Isolation of a Novel Antibacterial Phenyl Thioketone from the Seagrass, *Cymodocea serrulata*. Phytother Res. 2015; 29: 554-560.
- Grignon-Dubois M, Rezzonico B. First Phytochemical Evidence of Chemotypes for the Seagrass *Zostera noltii*. Plants. 2012; 1: 27-38.
- Grignon-Dubois M, Rezzonico B, Alcoverro T. Regional scale patterns in seagrass defences: Phenolic acid content in *Zostera noltii*. Estuar Coast Shelf Sci. 2012; 114: 18-22.
- Grignon-Dubois M, Rezzonico B. The economic potential of beach-cast seagrass – *Cymodocea nodosa*: a promising renewable source of chicoric acid. Botanica Marina. 2013; 56 (4): 303-311.
- Harborne JB, Williams CA. Occurrence of sulphated flavones and caffeic acid esters in members of Fluviales. Biochem Syst Ecol. 1976; 4: 37-41.
- Harrison PG. Control of microbial growth and of amphipod grazing by water-soluble compounds from leaves of *Zostera marina*. Mar Biol. 1982; 67: 225-230.
- Iyapparaj P, Revathi P, Ramasubburayan R, Prakash S, Palavesam A, Immanuel G, Anantharaman P, Sautreau A, Hellio C. Antifouling and toxic properties of the bioactive metabolites from the seagrasses *Syringodium isoetifolium* and *Cymodocea serrulata*. Ecotoxicol Environ Saf. 2014; 103: 54-60.
- Kontiza I, Stavri M, Zloh M, Vagias C, Gibbons S, Roussis V. New metabolites with antibacterial activity from the marine angiosperm *Cymodocea nodosa*. Tetrahedron. 2008; 64: 1696-1702.
- Kontiza I, Abatis D, Malakate K, Vagias C, Roussis V. 3-Keto steroids from the marine organisms *Dendrophyllia cornigera* and *Cymodocea nodosa*. Steroids. 2006; 71: 177-181.
- Kontiza I, Vagias C, Jakupovic J, Moreau D, Roussakis C, Roussis V. Cymodienol and cymodiene: new cytotoxic diarylheptanoids from the sea grass *Cymodocea nodosa*. Tetrahedron Lett. 2005; 46: 2845-2847.
- Laabir M, Grignon-Dubois M, Masseret E, Rezzonico B, Soteras G, Rouquette M, et al. Algicidal effects of *Zostera marina* L. and *Zostera noltii* Hornem. extracts on the neuro-toxic bloom-forming dinoflagellate *Alexandrium catenella*. Aquat Bot. 2013; 111: 16-25.
- Mani AE, Bharathi V, Patterson J. Antibacterial Activity and Preliminary Phytochemical Analysis of Sea Grass *Cymodocea rotundata*. Int J Microbiol Res. 2012; 3 (2): 99-103.
- McMillan C, Zapata O, Escobar L. Sulphated phenolic compounds in seagrasses. Aquat Bot. 1980; 8: 267-278.
- Mezghani S, Csupor D, Bourguiba I, Hohmann J, Amri M, Bouaziz M. Characterization of Phenolic Compounds of *Ulva rigida* (Chlorophycae) and Its Antioxidant Activity. European J Med Plants. 2016; 12 (1): 1-9.
- Popov SS, Marekov NL, Konaklieva MI, Panayotova MI, Dimitrova-Konaklieva S. Sterols from some Black Sea Ulvaceae. Phytochemistry. 1985; 24: 1987-1990.
- Quackenbush RC, Bunn D, Lingren W. HPLC determination of phenolic acids in the water-soluble extract of *Zostera marina* L. (eelgrass). Aquat Bot. 1986; 24: 83-89.
- Ragupathi Raja Kannan R, Arumugam R, Iyapparaj P, Thangaradjou T, Anantharaman P. In vitro antibacterial, cytotoxicity and haemolytic activities and phytochemical analysis of seagrasses from the Gulf of Mannar, South India. Food Chem. 2013a; 136: 1484-1489.
- Ragupathi Raja Kannan R, Arumugam R, Thangaradjou T, Anantharaman P. Phytochemical constituents, antioxidant properties and *p*-coumaric acid analysis in some seagrasses. Food Res Int. 2013b; 54: 1229-1236.
- Ravikumar S, Syed Ali M, Anandh P, Ajmalkhan M, Dhinakaraj M. Antibacterial activity of *Cymodocea serrulata* root extract against chosen poultry pathogens. Indian J Sci Technol. 2011; 4 (2): 98-100.
- Ravn H, Pedersen MF, Borum J, Andary C, Anthoni U, Christophersen C, et al. Seasonal variation and distribution of two phenolic compounds, rosmarinic acid and caffeic acid, in leaves and roots-rhizomes of eelgrass (*Zostera marina* L.). Ophelia. 1994; 40: 51-61.
- Sica D, Piccialli V, Masullo A. Configuration at C-24 of sterols from the marine phanerogames *Posidonia oceanica* and *Cymodocea nodosa*. Phytochemistry. 1984; 23: 2609-2611.
- Sun X, Jin H, Zhang L, Hu W, Li Y, Xu N. Screening and isolation of the algicidal compounds from marine green alga *Ulva intestinalis.* Chin J Oceanol Limnol. 2016; 34 (4): 781-788.
- Todd J, Zimmerman RC, Crews P, Alberte RS. The antifouling activity of natural and synthetic phenolic acid sulfate esters. Phytochemistry. 1993; 34: 401-404.
- Trigui M, Gasmi L, Zouari I, Tounsi S. Seasonal variation in phenolic composition antibacterial and antioxidant activities of *Ulva rigida* (Chlorophyta) and assessment of antiacetylcholinesterase potentiel. J App Phycol. 2013; 25: 319-328.
- Vergeer LHT, Develi A. Phenolic acids in healthy and infected leaves of *Zostera marina* and their growth-limiting properties towards *Labyrinthula zosterae*. Aquat Bot. 1997; 58: 65-72.
- Wang J, Pan X, Han Y, Guo D, Guo Q, Li R. Rosmarinic acid from eelgrass shows nematicidal and antibacterial activities against pine wood nematode and its carrying bacteria. Mar Drugs. 2012;10: 2729-2740.
- Zapata O, McMillan C. Phenolic acids in seagrasses. Aquat Bot. 1979; 7: 307-317.
